# Carbon-Centered Radicals in Protein Manipulation

**DOI:** 10.1021/acscentsci.3c00051

**Published:** 2023-03-21

**Authors:** Xuanxiao Chen, Brian Josephson, Benjamin G. Davis

**Affiliations:** † Department of Chemistry, University of Oxford, Oxford, OX1 3TA, U.K.; ‡ The Rosalind Franklin Institute, Oxfordshire, OX11 OFA, U.K.; § Department of Pharmacology, University of Oxford, Oxford, OX1 3QT, U.K.

## Abstract

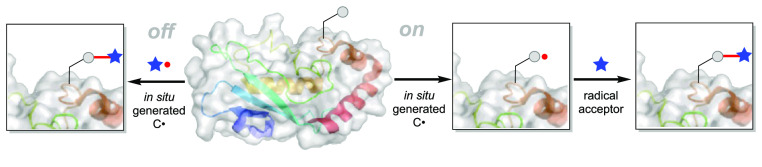

Methods to directly post-translationally modify proteins are perhaps the most straightforward and operationally simple ways to create and study protein post-translational modifications (PTMs). However, precisely altering or constructing the C–C scaffolds pervasive throughout biology is difficult with common two-electron chemical approaches. Recently, there has been a surge of new methods that have utilized single electron/radical chemistry applied to site-specifically “edit” proteins that have started to create this potential—one that in principle could be near free-ranging. This review provides an overview of current methods that install such “edits”, including those that generate function and/or PTMs, through radical C–C bond formation (as well as C–X bond formation via C• where illustrative). These exploit selectivity for either native residues, or preinstalled noncanonical protein side-chains with superior radical generating or accepting abilities. Particular focus will be on the radical generation approach (on-protein or off-protein, use of light and photocatalysts), judging the compatibility of conditions with proteins and cells, and novel chemical biology applications afforded by these methods. While there are still many technical hurdles, radical C–C bond formation on proteins is a promising and rapidly growing area in chemical biology with long-term potential for biological editing.

## Abstract 2

The alternative selectivity of C• radical chemistry can now be applied, both “off-protein” and “on-protein”, to selectively “edit” proteins in a useful manner that circumvents the limits of common two-electron approaches.

## Introduction

As the primary workhorse biomolecules, responsible for a multitude of critical functions within biology, understanding the function of proteins on a molecular level is of extreme interest in many research fields. They are often extensively diversified by post-translational modifications (PTMs), with potential to greatly increase their functional breadth.^1^ Further propelled by their association with many pathologies, and their promise as therapeutics,^2^ it is not surprising that a plethora of methods to chemically and site-specifically modify proteins have been developed with the purpose of both mimicking native residues and PTMs or attaching useful chemical handles for a broad range of applications.^3−5^ Recent advances, exhibiting a diverse range of applications, have been successfully illustrated in targeted drug delivery,^6^ specific cellular tracking technologies,^7^ and fluorescent imaging.^8^ These have also highlighted that a remaining challenge is one of precision in chemistry. In order to recreate native, modified (or even unnatural) residues with programmed, understood function there is a need to generate them without causing disruption to innate activity or structure. Novel methods for the site-selective “scarless” or “traceless” installation of useful tags that furnish homogeneous protein adducts, allowing study in functional or native contexts, are therefore in high demand.

The field of protein chemistry can be summarized as a quest for chemical selectivity at several levels. One of the main challenges facing chemoselective protein modification methodologies is the difficulty in achieving selective reactivity preferentially with the targeted residue of interest, in a “sea”^9^ of other side-chains and biogenic functionality. To fulfill this criterion, many methods target the amino acids that have lower abundance (low “copy number”) on the protein surface and possess unique physiochemical properties, facilitating a distinct reactivity over other common residues. Although continuous interest has been shown in targeting Lys, Cys, and to a lesser extent other less reactive or nucleophilic residues,^10^ with their nucleophilic heteroatoms allowing for reaction with off-protein electrophiles, the specific targeting of one residue to afford an homogeneous adduct still remains a challenge in the field. As a result, many bioconjugates are still made and used as mixtures.^11,12^ In addition to selectivity issues, the reagents and conditions must ensure a benign environment to preserve the native structure and function of the protein. In most cases relevant chemistry requires near-physiological pH (pH ≈ 7) and temperature (*T* ≤ 37 °C), as well as (ideally fully) aqueous media, low reagent concentrations (low mM and below), and short reaction times (<1 h). A failure to address these requirements still plagues several (often innovative) peptidic chemical methods that, due to a requirement for, e.g., organic solvents or high substrate concentrations, remain centered in an impractical regime with limited adoption in biology. Yet, potential for new methods remains high.

A now relatively common approach to create site-specific protein modifications via low copy number functional groups is based on alternative codon use^13^ such as amber-codon suppression.^14,15^ The latter genetic engineering method allows the incorporation of noncanonical amino acids (ncAAs) consisting of many PTMs and unnatural side-chain variants, into proteins during translation. Functionalities such as azides,^16^ alkynes,^17^ halogens,^18^ and ketones^19^ have proven popular as they can participate in further reactions that are useful even in testing biological contexts.^20^ For example, reagents exploited in the commonly used (and now celebrated) strain-promoted azide–alkyne [3 + 2] cycloaddition^21^ possess only low level reactivity with surrounding biogenic functional groups^22,23^ and so combine well with this approach.^24^ However, while utility in living systems remains powerful^15^ it may be the case that, despite this strategic elegance, other considerations (e.g., quantities of necessary ncAAs, sometimes low incorporation efficiencies in expression hosts,^25^ off-target incorporation into other amber codon sites,^26,27^ and incompatibility between the desired ncAA and the orthogonal tRNA/tRNA synthetase pair) could prove translationally prohibitive in other scenarios, such as the sustainable large-scale production of novel and synthetic biologics, for example.

Therefore, strategies focused on direct protein modification of native systems or natively derived systems remain attractive. Among the successful methods for selective functionalization of native residues, those that involve heterolytic/2e- transformations remain understandably dominant.^3^ Yet, for example, construction of a framework prevalent in nature—the C(sp^3^)-C(sp^3^) bond—is essentially out of scope for such methods. As a consequence, protein modification methods have instead typically focused on exploitation of heteroatom (X−X or X−C) chemistry, and, indeed, this is the near exclusively dominant strategy in bioconjugation.^4,28^ In essence, the field has focused on synthetic challenges that *can* be addressed but has perhaps unnecessarily limited itself by not considering more directly those that *should* be addressed.

C(sp^3^)–C(sp^3^) bonds are highly abundant throughout most biomolecules, including the side-chains of proteins. They are difficult to form with the cationic or anionic synthons utilized in traditional two-electron chemistry while retaining benign, biocompatible conditions and site-/chemoselectivity. As an alternative, a growing number of chemical biologists are utilizing the unique reactivity exhibited by single-electron carbon-centered C• species in “open-shell”/radical methods to expand the toolkit available for chemical manipulation of proteins.^28,29^ Despite radical reactions having the reputation of possessing an uncontrollable reactivity in biology, synthetic chemists have long showcased many reliable radical reactions in aqueous media,^30^ raising the potential for their application in nature. Additionally, many organic (including, critically, carbon-centered C•) radical intermediates have demonstrated utility and potential biocompatibility in biosynthesis, as exemplified by the diverse manifolds mediated by radical S-adenosylmethionine enzymes,^31^ some with relatively high stability in aqueous buffers at ambient temperatures. This directly highlights their synthetic utility in biology.

During the last two decades, several reviews have emphasized the destructive role of reactive oxygen species (ROS), such as hydroxide and superoxide radicals, in the pathophysiology of many neurodegenerative diseases^32^ and cancers.^33^ Indeed, these species can cause acute damage when they covalently react with a given protein.^34^ As a consequence of this oxidative damage, the prerequisite for useful “open-shell”/radical-mediated protein modification methods is to avoid ROS. Yet, the use of radicals via chemistry that avoids the extreme redox potentials associated with ROS may in fact remain a powerful and selective avenue.^35^

**Figure 1:**
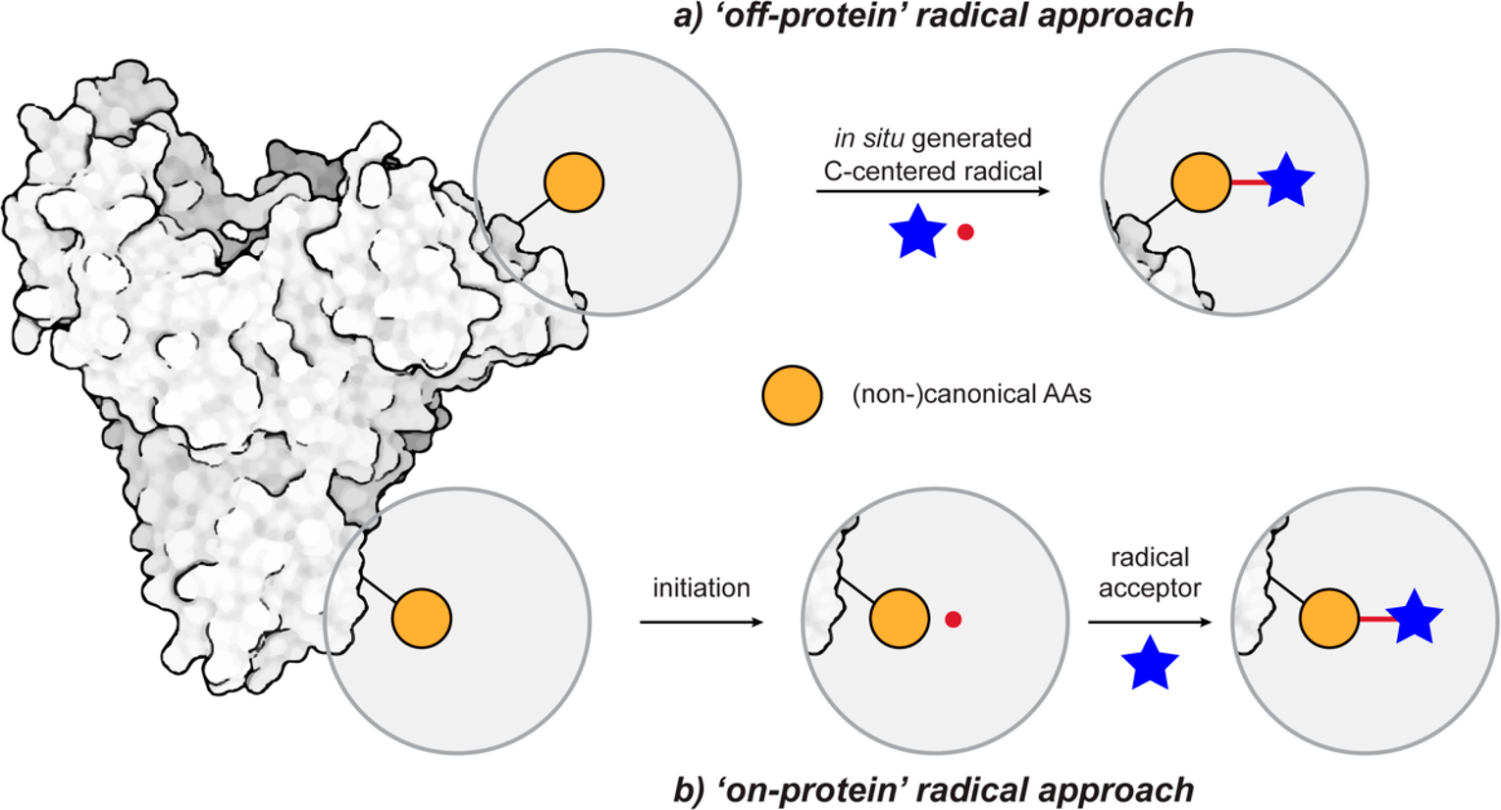
(a) General scheme for “off-protein” radical approach. (b) General scheme for “on-protein” radical protein modification.

Simplistically, one can devise two different radical-mediated strategies employed for selective protein modification. One is an “off-protein” radical approach (Figure 1a), where radicals are generated from external radical precursors that then react selectively with the protein, ideally at a specific residue of interest. The other strategy involves an “on-protein” radical approach (Figure 1b), relying on direct radical generation from natural or unnatural residues in proteins, followed by subsequent trapping with external radical acceptors/partners. In our opinion, organic chemists have proposed numerous promising methods for preparing radical precursors, but the requirement for both mildness and biocompatibility (see above) in their activation to initiate reactions has prevented many of them from being applied to protein modification.

Biomimetic radical modification processes, such as the now near-classical use of tyrosinyl-derived radicals generated from oxidation using peroxidase and H_2_O_2_,^36^ provide valuable strategic illustrations. In this way, bioinspired/biomimetic radical-mediated protein-labeling methods were presciently demonstrated^37,38^ (notably with methods that would now be described as so-called “photoredox” catalysis) and then further explored with diverse modes of oxidative radical generation, including enzymatic, photochemical, and electrochemical processes.^39^ Therefore, while pathways reliant on SET to or from target substrates from the excited state of a catalyst activated *in situ* upon irradiation with light have been highlighted in the context of their potential mildness and wide applicability,^40^ these methods should perhaps be considered as just one of the several ways in which substrates can be either oxidized or reduced to generate powerful carbon-centered C• radical intermediates to furnish subsequent functionalization.

In this review, we aim to highlight the key developments toward biocompatible radical-based protein modification methodologies, focusing on chemically generated carbon-centered C• radicals, with discussion focused on current limitations as well as future directions. As posited above, we employ an “off-protein” vs “on-protein” delineation (Figure 1); for approaches employing cross-coupling between “off-protein” radicals and “on-protein” radicals, they have been classified as an “on-protein” radical approach since the selectivity is greatly governed in these instances by the on-protein site.

With an eye toward the need for relevance discussed above, this review will also focus primarily on methods applicable to proteins. However, methods that employ peptides that possess sufficient length and secondary structures can prove a reasonable mimic of protein complexity, and as such, where relevant, will also be covered. The potential of certain methods currently restricted to peptides will also be considered for their promise.

## “Off-Protein” Radical Approaches

The “off-protein” radical approach (Figure 1a) exploits specific AAs in proteins to act as radical traps (“SOMOphiles”) or partners with external carbon-centered C• radicals. To fine-tune radical generation conditions, and in the context of the compatibility constraints discussed above, certain central requirements exist:

1) radical generation/initiation processes prove mild and biocompatible, sufficient to not disrupt protein function;
2) the generated radical species needs to be relatively stable (sufficiently long-lived) to allow trapping by the target residue;
3) no (or minimal) ROS species should be generated throughout the modification processes.

Some of these constraints may be usefully considered in the context of redox windows (and the corresponding half-potentials for single electron transfers that may drive initiation or radical quenching) that are compatible with biology^35^ and indeed water^41^ to avoid direct and indirect damage.

In some cases, the reactivity of resulting radicals with corresponding SOMO-philes should also be considered. For example, several current examples in protein modification are primarily based on “off-protein” C• sp^3^ hybridized radicals. Simple aryl (C• sp^2^) radicals are more rarely seen and prove less tractable, something that may be attributed, in part, to the known associated larger C• SOMO-to-acceptor LUMO gaps in certain cases.

Despite potential limitations, thanks to the large versatility of off-protein radical precursors, various functionalities can be installed on proteins through such a strategy, thereby giving this approach a broad scope.^28,35^ Given this diversity and the critical role of the protein functional group that acts as a SOMO-phile acceptor, these “off-protein” radical approaches will be classified into two categories, based on the qualities of that target radical acceptor. These are also split according to the strategy for their use as either inherent/endogenous *or* those purposefully created as reaction “tags”:^42^ that is, radical additions to canonical AAs, *or* radical additions to noncanonical AAs (ncAAs). Furthermore, the methods will be evaluated semicomprehensively in terms of their operational requirements and their applicability to site-selectively functionalize their target residues.

### Radical Additions to Canonical AAs

**Figure 2:**
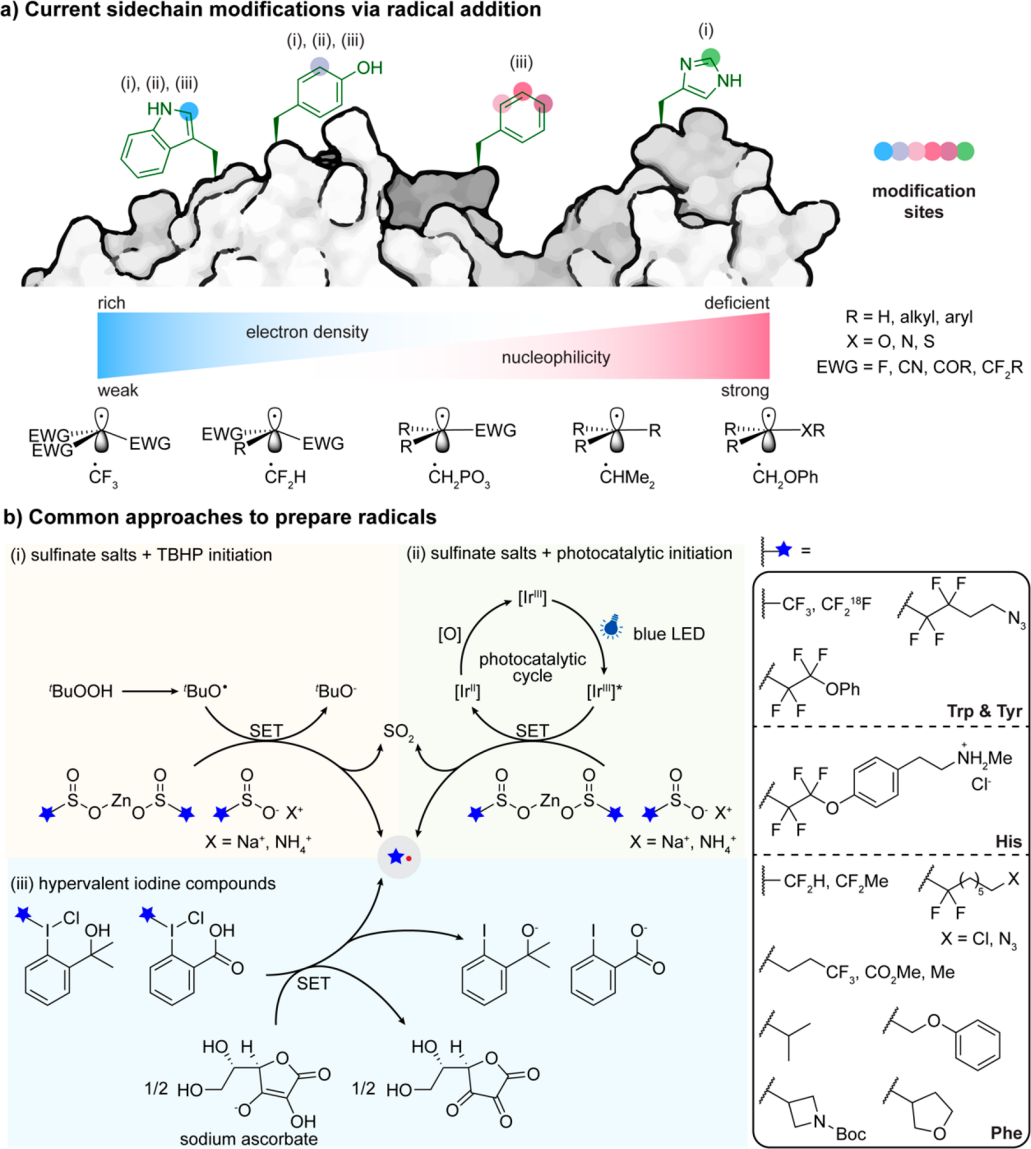
a) Overview of current side-chains that can be attached via radical additions to canonical AAs. b) Selected mechanisms for some common approaches to “off-protein” carbon-centered C• radicals with utility in reactions with canonical AAs. These include (i) sulfinate salts in conjunction with direct oxidant^43−45^ e.g. TBHP; (ii) sulfinate salts in conjunction with photocatalytic oxidant;^46^ and (iii) hypervalent iodine salts in conjunction with ascorbate salts.^47^

From an experimental perspective, the modification of canonical AAs is immediately beneficial in that no pre-engineering of the target protein is necessary. As a result, possible yield losses during extra steps needed to install a reactive handle can be largely avoided, resulting in an overall more straightforward approach. As a result, there has been a continuous interest for protein labeling via canonical residues,^10^ with the majority of research largely confined to aromatic AAs as SOMOphiles, regardless of the types of “off-protein” radicals that have been tested. This is unsurprising as a principal reaction pathway in this approach follows radical additions to unsaturated systems. In this way the electronic properties of different residues guide their reactivity profile toward different radicals (Figure 2a).

**Figure 3:**
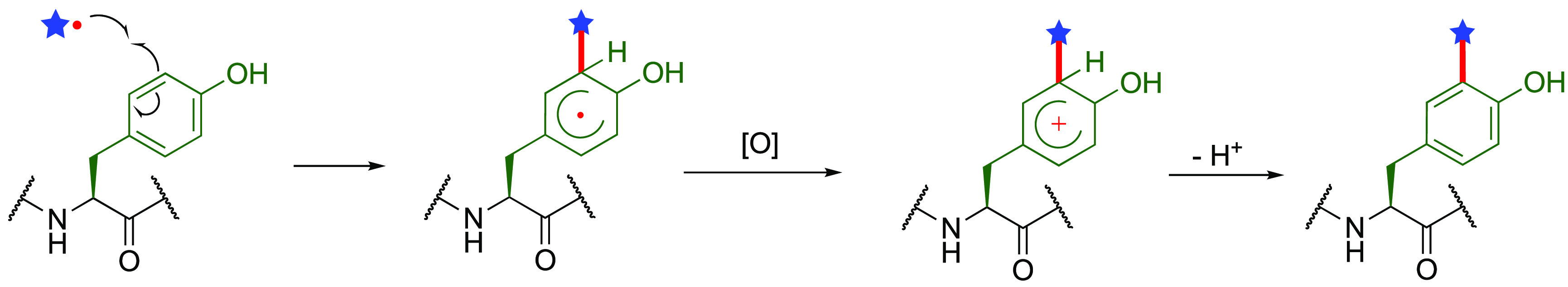
Suggested mechanism of Tyr-selective peptide functionalization using off-protein radicals, generated from sulfinate salts.^46^

**Figure 4:**
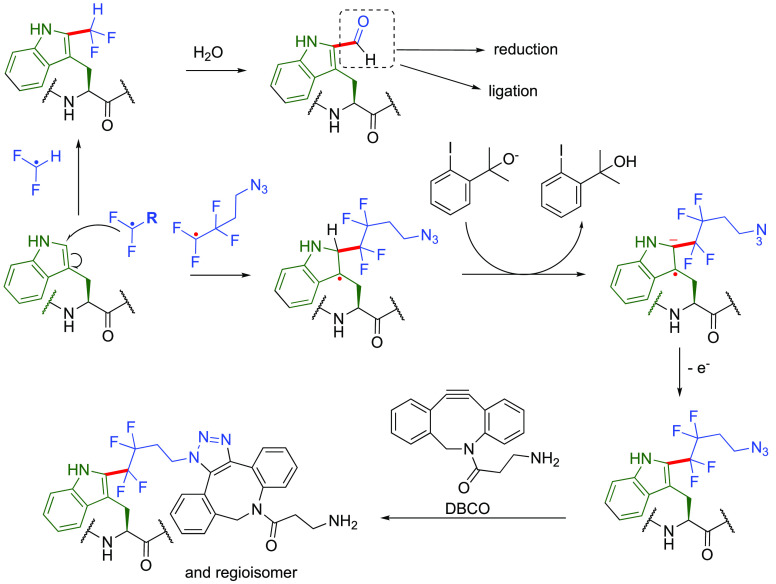
Pathways and mechanisms of Trp-selective protein functionalization using off-protein •CF_2_R radicals. Reaction at C2 gives a range of modified protein adducts, including variants that react further spontaneously (e.g., via hydrolysis to formyl, top^43^) or via additional e.g. dibenzocyclooctyne (DBCO) derivatives (bottom).^47^ A suggested mechanism from hypervalent iodine compounds is shown here (bottom, adapted from ref (47)).

The electron-rich characteristic of certain aromatic native residues, such as Trp and Tyr, predispose them to react with electrophilic radicals (Figures 2, 3, and 4), something that may be attributed in part to lowered SOMO energies.^48^ The corollary is that more nucleophilic radicals, such as hydrocarbon-based radicals, are less prone to react with these residues.^49^ As a consequence, the strategies presented here can be categorized into three broader groups: radical additions to *electron-rich*, *electron-deficient*, or *other* aromatic AAs.

#### Electron-rich Aromatic Canonical AAs – Trp and Tyr

As noted in the Introduction, one dominant mode of reactivity when considering C• centered radicals is the use of peroxidase-mediated generation of phenoxyls. While the utility of tyrosinyl in proteins (see below in “on protein”) can be considered, the generation and use of phenoxyls as modifiers of Tyr in often high copy number has been particularly (and ingeniously) exploited in protein mapping. A dominant methodology has been that of the APEX^50^ system that permits use even in complex environments. This exploits hydrogen peroxide as an oxidant and designed peroxidase constructs (or fusion systems) as the catalysts for radical formation that diffuse to react with local targets. This approach has, in turn, triggered consideration of numerous processes that now exploit similar localization methods for enhancing partner modification (an area that in some contexts has recently become known as proximity-dependent labeling (PDL)^51^). The APEX and associated systems are often used to deliver a label via the phenoxyl (e.g., biotin) allowing tracking and affinity-based retrieval of products. They have been excellently reviewed elsewhere^52^ and will not be covered here again.

According to one analysis of reactivity profile, electrophilic radicals may react preferentially with electron-rich acceptors, concomitant with progressively increasing electrophilic nature as, for example, the number of incorporated fluorine atoms increases.^53^ Prompted by the importance of fluorine-containing motifs (Figure 2a) in diagnostics and medicinal chemistry, there has therefore been a growth of interest in modifying biomolecules with various fluorine containing tags.^46^ Therefore, fluorinated alkyl radicals, such as •CF_3_, prove useful not only in their application but also in their ability to undergo reactions with electron-rich canonical AAs such as Trp and Tyr.

This potential has been realized in proteins through use of a series of mild reagents and/or conditions that allow SET under protein compatible scenarios to fluoroalkylate proteins. Substituted sulfinate salts are one of the most representative sources of fluoroalkyl radicals employed in this regard. Langlois’ reagent (NaTFMS)^54^ and salt-altered variants (e.g., ZnTFMS)^55^ have proven popular as oxidatively initiated sources (Figure 2b). Thus, with the help of organic oxidants, photochemistry, or even electrochemistry,^56^ these sulfinate salts can undergo single-electron oxidation to produce corresponding fluoroalkyl radicals, with concomitant release of sulfur dioxide.

Functionally, addition of such radicals to Trp and Tyr has the potential to allow for label-enabled interrogation including protein-observed ^19^F-NMR spectroscopy. This can be highly sensitive and responsive to the local environment and covers a broad chemical shift (and so functional group) range.^57^ Additionally, fluorine-mediated methods possess zero background, due to the essentially complete lack of fluorine in relevant nature. With the help of this technique, protein structures and functions, as well as interactions with ligands, can therefore be considered.^58^

As noted above, initiation conditions provide a major potential constraint for off-protein C• radicals. *tert*-Butyl hydroperoxide (TBHP) is an organic oxidant commonly used as a radical initiator (Figure 2b). However, within the context of protein modification, an additional sacrificial reductant may be required to ameliorate potential oxidative side-reactions on other residues (or even the starting material). Based on such a strategy, selective and benign trifluoromethylation can be accomplished in proteins.^43,44^ These reactions (200–300 equiv NaTFMS, 12.5–25 equiv TBHP, 25–50 equiv Met, 5–10 min, 0 °C, pH 6.0) reveal that the C2 position of Trp is the preferred trap among all other canonical sites in proteins (Figure 2a). Application of the method to several representative protein substrates, including myoglobin and lysozyme, confirmed that these conditions do not significantly alter the native function of proteins, even in the presence of reductively sensitive disulfide bridges.^44^ Notably, slightly acidic pH (6.0) promoted trifluoromethylation 30-fold faster at Trp compared with other possible modification sites, including His, Tyr, and Cys. Moreover, the inherently “small” nature of the reactive intermediates generated ensured good “reactive accessibility”^59,60^ to target even internal/core Trp residues. This, in turn, enables valuable “sensing” of substrate engagement, even in for example quite deep active sites, via ^19^F-NMR. Such methods also prove to be sufficiently robust to allow ^18^F-containing sulfinate salts to be used, enabling biomolecule radiolabeling, with potential utility in positron emission tomography (PET).^45^ The lack of requirement for prefunctionalization of biomolecules thus suggests a strategy that may simplify the radiolabeling process. However, the difficulties arising from preparing ^18^F-incorporated sulfinate salts and lower molar activities should be noted as possible caveats.

While electrophilic radicals are seemingly necessary for good reactivity, there is sufficient latitude to allow extension of this reactivity to examples that use •CF_2_R. Notably •CF_2_H remains an intriguing case that following reaction at Trp appears to proceed through a Vilsmeier-like pathway leading to overall formylation at C-2 (Figure 4). Direct formylation is rare in protein methods—this not only provided access to a reactive functional group for carbonyl-selective transformations but in itself allowed modulation of Trp fluorescence through the formation of C-formyl-Trp (Cfw).^43^ Initial promise (requiring some control of conditions and reaction timing) was also shown for this method even for cell surface modification, observable in cellular populations via Cfw fluorescence.

These studies have revealed an electronic hierarchy that favors Trp as a useful, but also somewhat more rare, residue in proteins. However, lower but nonetheless useful reactivity with more common Tyr proves effective also. In this way labeling and even radiolabeling of human insulin via Tyr-selective trifluoromethylation (in the absence of Trp) proves feasible and effective ([^18^F]CF_3_SO_2_NH_4_ (20–30 MBq), 11.5 equiv TBHP, 5.8 equiv Fe(NO_3_)_3_·9H_2_O, 20 min, 40 °C),^45^ with the main target site being Tyr19 of the A-chain (Figure 3).

Other than using TBHP to generate radicals from such sulfinate fluorination reagents, Ir(lll)-based photocatalysts have also been reported to be effective in selectively installing fluorinated labels on several oligopeptides (Figure 2b). Thus, subsequent adaptation by the Merck Laboratories^46^ of this use of sulfinate sources has allowed several relevant peptides, including splenopentin, angiotensin, and dermorphin (Figure 3), to be targeted (15 mol % Ir[dFCF_3_ppy], 20 equiv NaTFMS, blue LED, 20 h, 26 °C). Notably, in addition to confirming that this analogous ^19^F-modification shares the same preference for the Tyr19 site of A-chain of human insulin (27 equiv NaTFMS, 50 equiv TBHP, 16 h, 30 °C) it also allowed valuable comparison of the differences between photoredox and direct oxidant (e.g., TBHP) initiation methods. Suggested benefits for the delivery of a catalytic amount of oxidant include mildness, reduced Tyr dimerization, and lower concomitant oxidation of Met. It should be noted that aspects of these latter strategies perhaps stretch the bounds of biocompatibility when applied to peptides (e.g., conditions as low as pH ≈ 2) to avoid some undesired modifications. Interestingly, these studies also note some encroaching reactivity with other aromatics such as Phe (of relevance below).

While methods for initiation of off-protein radicals can in principle be diverse, one set that has proven in practice complementary for electron-rich canonical AAs to the oxidative initiation of sulfinate salts is those that use hypervalent iodine compounds (Figure 2b) that can be reductively activated. These have also been commonly used as fluorination agents in organic chemistry. Transition metals have been employed to catalyze such reactions for small molecules,^61^ but the use of sodium ascorbate can prove protein compatible.^47^ Aryl-λ^3^-iodanes allowed selective installation of not only the CF_3_ moiety into oligopeptides (Figure 3), but perhaps more pertinently allowed some useful handles to be installed into biomolecules via substituted variants (10–200 equiv iodanes, 10–150 equiv sodium ascorbate, 1–15 min, 25 °C, pH 5.0–9.0). Thus, an azide moiety was introduced into a Y59-modified ubiquitin (in the absence of Trp). Consistent with the typical chemoselectivity profile, this method was highly selective for Trp when present; a Trp-modified adduct was observed for myoglobin under the same conditions (Figure 4). Again, driving such reactions at extremes highlights lower limits of selectivity. Other aromatic residues in carbonic anhydrase I were observed in this system when a large excess of reagent was employed.

#### Electron Poor Aromatic Canonical AAs – His

His displays altered reactivity when protonated; its imidazolium is slightly electron deficient giving rise to a preferential reaction at C2 toward more nucleophilic carbon-centered radicals. Carbon centered C• radicals, generated again using sulfinate salts with TBHP (Figure 2b), allowed productive addition reactions with a range of alkyl side-chains, including those with azetidine and ester substituents,^62^ (12 equiv substituted sulfinates, 20 equiv TBHP, 4 h, r.t. or 70 °C) in certain unprotected peptides. This promising mode of chemoselectivity might prove more challenging in more complex full-length substrates. Likely limitations could result from the relatively harsh conditions, i.e. strongly acidic conditions (pH ≈ 3) and high temperatures (up to 70 °C) required to drive the reaction and guarantee the selectivity of His over other residues.

**Figure 5:**
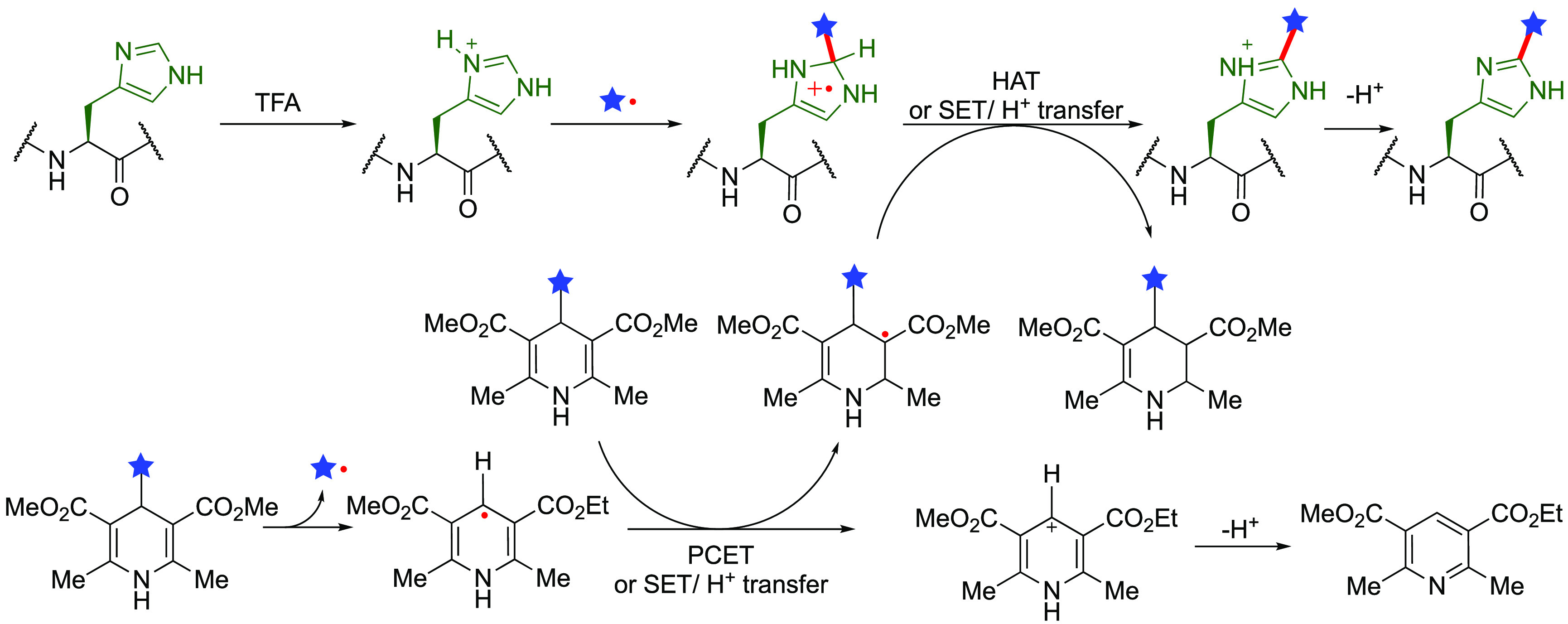
His-selective pathways exploiting imidazolium generation. Altered chemoselectivity, here using Hantzch-type precursors^63^ highlights the promise in certain systems but may restrict application to systems tolerant of lower pH. Figure adapted from ref (63). Copyright 2019 American Chemical Society.

His-selective reactions have also been demonstrated on longer peptides up to the 76-mer ubiquitin through the exploitation of a visible-light-mediated Minisci-type pathway using 4-alkyl-1,4-dihydropyridine (DHP)/Hantzsch-type reagents (Figure 5).^63^ The authors proposed that the DHP reagents served as both alkyl radical precursors and H atom-transfer acceptors. This is another promising example of altered selectivity that likely again hinges on the need for His protonation to form the imidazolium under the optimized conditions (10 equiv DHP, 10 equiv trifluoroacetic acid (TFA) in trifluoroethanol as solvent, 3 h for 3 rounds, 35 °C). It was shown that functionalized ubiquitin remains intact, and the avoidance of oxidant is a strong potential advantage. In some cases, the necessity for superstoichiometric quantities of TFA, the use of trifluoroethanol, and the incompatibility of the method with unprotected Cys may restrict its applicability in more complex protein systems.

#### Other Aromatic AAs – Phe

Beyond the more reactive indole, phenol, and imidazole motifs, the phenyl moiety in Phe has also been shown to act as a possible radical trap. However, given that it has no activating substituents, reactions on this residue tend to deliver limited selectivity, especially in the presence of other aromatic AAs. Some substrates with more limited functional group content may therefore provide feasible substrates. Radicals reductively generated from hypervalent iodine compounds in conjunction with sodium ascorbate (Figure 2b) allowed, for example, the selective modification of Phe in nonapeptide bradykinin,^47^ an unprotected oligopeptide containing only one type of aromatic AA (100 equiv iodanes, 100 equiv sodium ascorbate, 15 min, 25 °C, pH 7.0).

### Radical Additions to Noncanonical AAs

**Figure 6:**
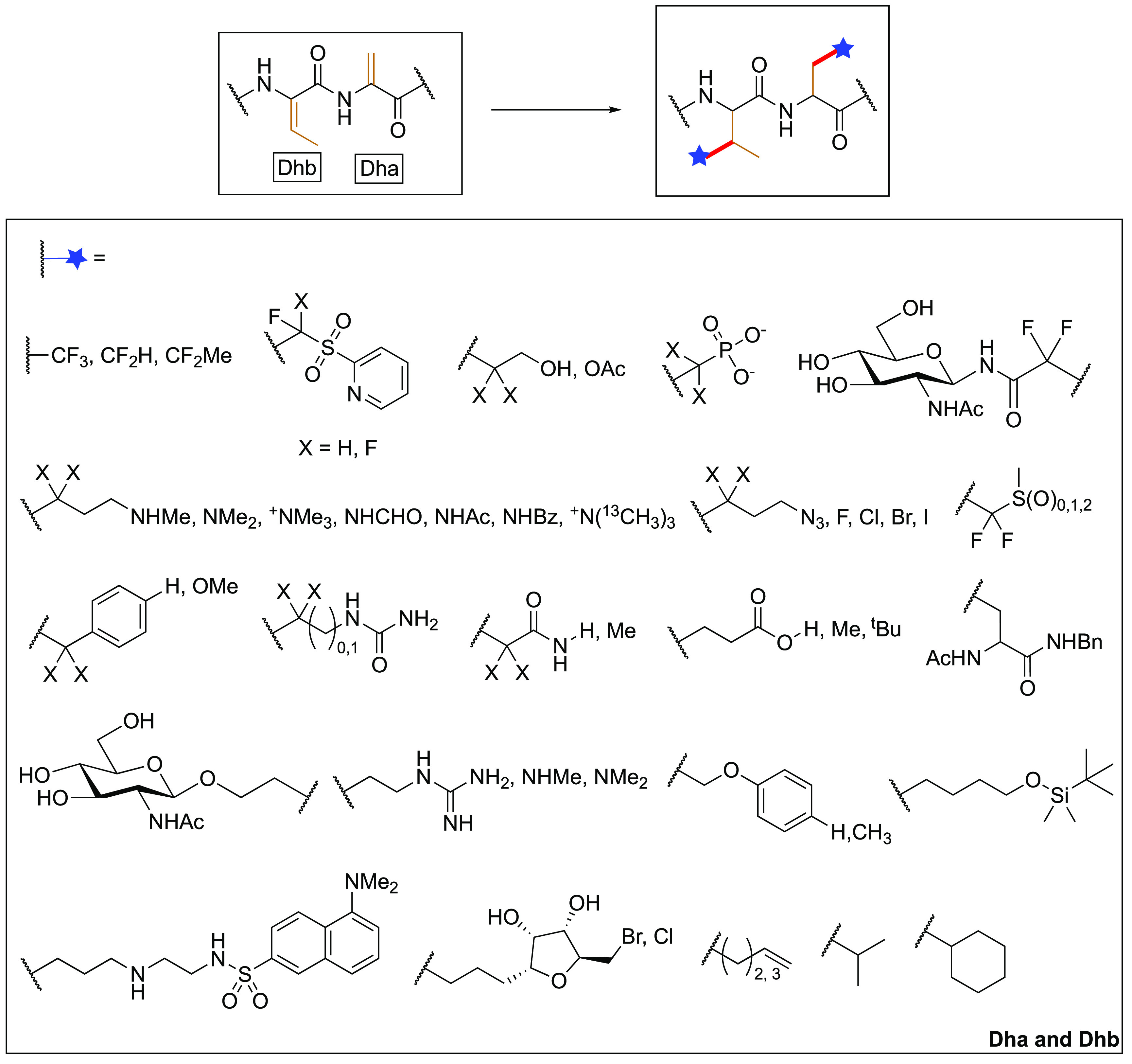
Overview of possible side-chains that can be attached via radical additions to noncanonical AAs in peptides and proteins.^28,29,35,64^

Since canonical AAs in biomolecules are relatively inefficient radical acceptors (SOMO-philes), the scope of functionalities that can be installed and the selectivities that can be achieved on native, unmodified proteins remain largely limited. To improve the radical accepting ability, dehydroalanine (Dha), an alkene-containing AA has emerged as a promising alternative (Figure 6).^65,66^ From an electronic perspective, Dha exhibits a high reactivity toward both electrophilic and nucleophilic radicals. This can be considered, in part, through SOMO(C•)-LUMO(Dha) transition states with extended interactions of the Cα=Cβ double bond with backbone Nα and C(O)α as electron-donating and electron-withdrawing substituents, respectively. Additionally, the α-carbon radical intermediate resulting from radical addition to Cβ is stabilized by the captodative effect.^67^

As a result, diverse modes of installation of this noncanonical AA, Dha, tag into proteins now exist. A wide variety of methods, exploiting both genetic code expansion and traditional site-directed mutagenesis to control site, unify around typically two-stage use, incorporation or generation of a Dha precursor (e.g., Cys, pSer, modified Sec), and then elimination (chemically or enzymatically). These are reviewed elsewhere,^68,65^ but the diversity of access strategies to this class of protein intermediate has seen useful adoption of many subsequent Dha chemistries. In this way, this promising method has effectively expanded the toolbox available for late-stage functionalization of proteins. Notably, the (re)generation of a stereogenic center at Cα in the protein backbone in these methods proceeds with typically poor stereoselectivity and so results in d-/l- mixtures of the resulting modified AA.

The first application of radical additions to Dha in protein substrates was independently reported by both our group^28^ and others,^29^ demonstrating a wide scope of off-protein radical precursors prepared from alkyl halides. One approach uses sodium borohydride (to initiate and/or quench radical intermediates) from alkyl bromides and iodides, followed by trapping with the preinstalled Dha to allow site-specific editing of proteins^28^ (100–2000 equiv alkyl halides, ∼1000–9000 equiv sodium borohydride, 30 min, 4 °C, pH 4.0–8.0). It is noteworthy that the side-chains installed, ranging from isotopically labeled structures to complex glycans, have enabled this methodology to recreate or mimic many essential PTMs on proteins, including methylation, acetylation, phosphorylation, and even glycosylation through the on-protein formation of Cβ–Cγ to a range of side-chain motifs. Mechanistic analysis suggested that control of oxygenation levels in aqueous solution (∼<6 ppm) and preferred suitability of borohydride over metal-initiated methods (including zincates) both prove important to control side-reactions. Concurrently, Park and co-workers focused only on metal-mediated methods to prepare radicals from alkyl iodides.^29^ Interestingly, zinc-organocopper derivatives proved apparently suitable (300 equiv alkyl iodide, 300 equiv zinc powder, 100 equiv Cu(ll) salts, 30 min, r.t., pH 4.5) for installation of modified Lys (methyl-, formyl-, and acetyl- Lys PTMs). Both approaches revealed excellent chemoselectivity and hence site-specificity at the Dha tag, as well as regioselectivity for Cβ, even for protein substrates with many aromatic residues. Synthetic proteins generated with the borohydride method also retained their structure as well as diverse function e.g. enzymatic activity and antibody-binding.^28^

**Figure 7:**
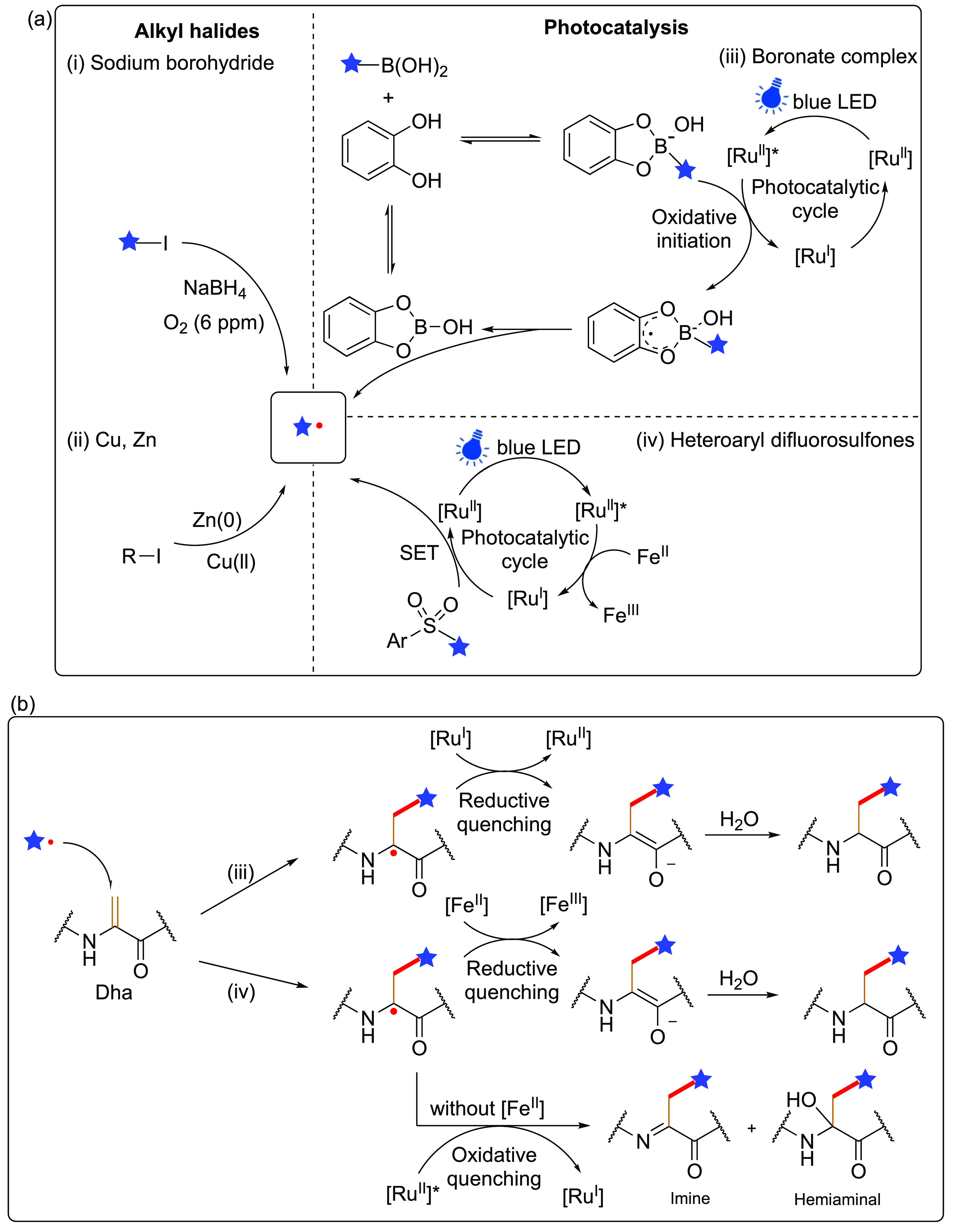
Suggested mechanisms for preparing and using off-protein radical precursors for additions to noncanonical AA Dha. (a) Generation of radicals using (i) alkyl halides in conjunction with sodium borohydride,^28,69^ (ii) alkyl halides in conjunction with Zn(0) or Cu(ll)/Zn(0),^28,29^ (iii) organoborates^35^ and (iv) heteroaryl difluorosulfones.^35,70^ (b) Suggested associated mechanisms for radical quenching steps of on-protein Cα• radical intermediates in two photocatalytic approaches.^35^

To further expand the applications in protein functionalization, a desire to incorporate alternative or reactive chemical handles (including side-chains containing redox sensitive functional groups) has led to further refinements in the conditions used and associated function that may be created in proteins. Evaluation of diverse photostimulated redox catalysts as initiators led, again, to a focus on biocompatibility that proved pertinent. More mild, protein-compatible Ru(ll)-based systems proved effective under two sets of complementary conditions for both reductive and oxidative initiation, depending on the radical precursor. Oxidative initiation (accessed via the reductive Ru(II)/(I) quenching cycle of Ru(ll)*) was shown to efficiently generate carbon-centered radicals from alkylboronate complexes, benefiting from an *in situ* generated boronic acid catechol ester displaying lowered oxidation potentials than the alkylboronates alone.^35^ This oxidative approach tolerated an unprecedented variety of chemical functionalities, such as halogen, azide, and ester containing substrates. Concurrently, reductive initiation in the presence of Fe(II) also allowed use of substituted heteroaryl difluorosulfones,^71^ generating difluoroalkyl carbon-centered radicals (and hence access to fluorinated side-chain variants also). Observation of products consistent with on-protein imine intermediates under certain conditions in the absence of coreductant led to the proposal that Fe(ll) salts may not only drive initial reduction of Ru(II)* to Ru(I) but also reductive quenching of intermediate on-protein α carbon radicals (Figure 7b).

**Figure 8:**
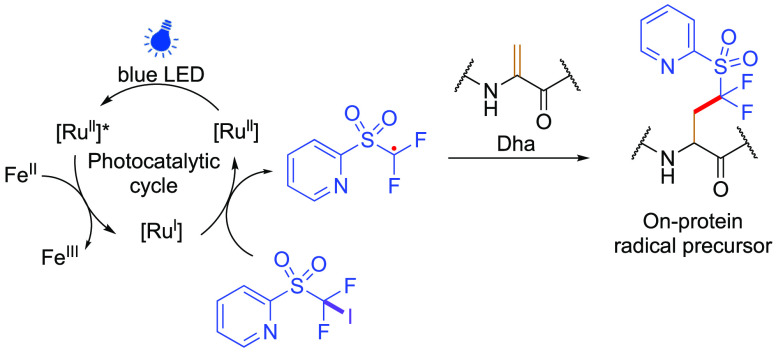
Proposed mechanism and process for installing an on-protein radical precursor via chemoselective reductive initiation through C–I homolysis in the presence of potentially labile C–S (which can then act later as a site for “on-protein” initiation, see below).^35^

Notably, use of dual heteroarylsulfone-iodide-substituted precursors also allowed selective sequential chemoselective reductive initiation of, first, the iodide (via C–I homolysis) and then the heteroarylsulfone (via C–S homolysis) (Figure 8). In this way, through this combination of controlled, sequential initiations the heteroaryl sulfones could, first, be installed into proteins and then, second, initiated as “on-protein” radicals (see also Figure 20 in the section “On-Protein” Radicals Generated on Noncanonical AAs for more detail). In this way, protein compatible radical precursors could be generated in protein side-chains for the later use of “on-protein” radicals.

**Figure 9:**
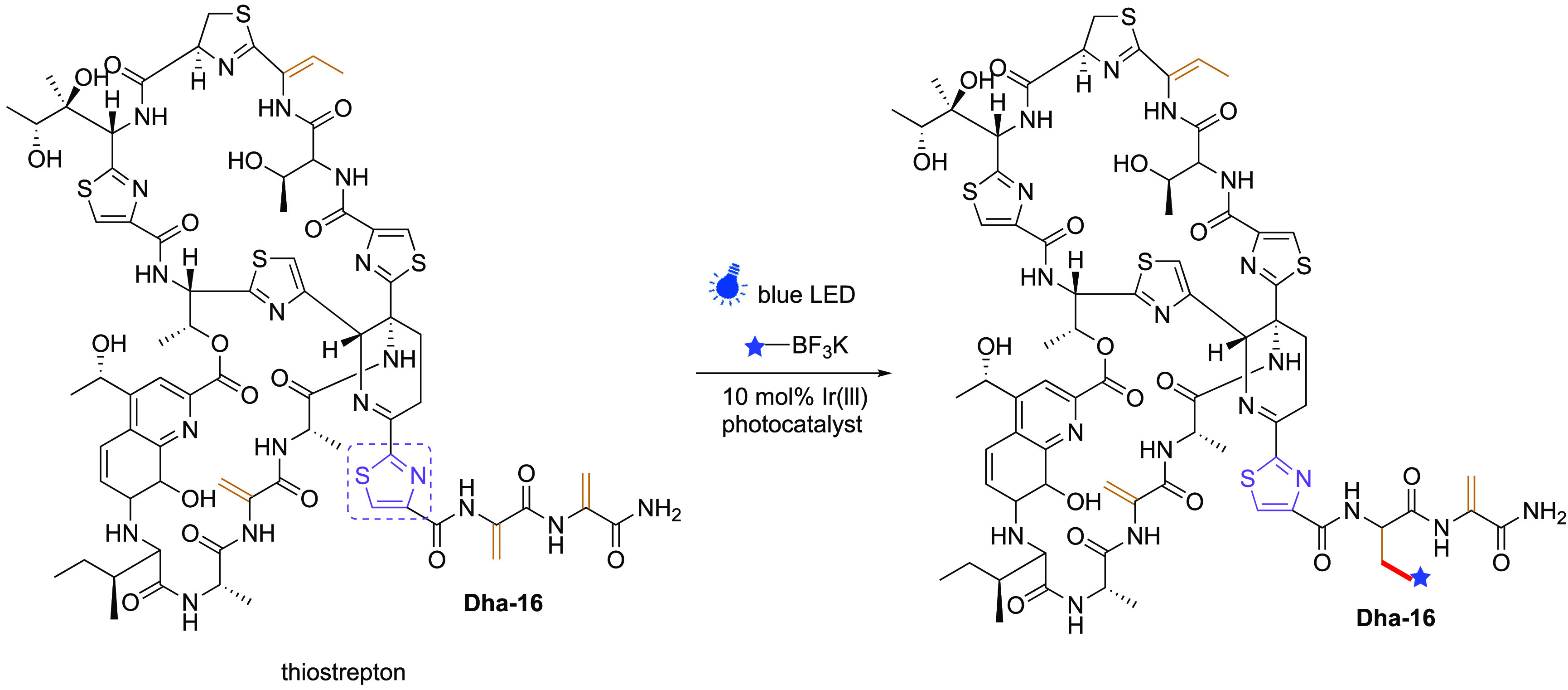
Regio- and chemoselective reaction of radicals with peptide natural product thiostrepton. The electron withdrawing effect of the thiazole group (purple) is suggested as a guiding motif for selective reactivity; how this will relate to utility in proteins using “off-protein” C• centered radicals remains to be seen, but it implies intriguing potential.^64^

While these methods rely upon the strong SOMO-philicity of Dha the potential for chemoselectivity over Dhb can be considered, given the typically strong “alpha steric effect”^72^ of the carbon undergoing attack. In this context, valuable information can be gained from peptidic systems even if using conditions (“strong” redox photocatalysts, precursors with high *E*_ox_, solvents, higher concentrations) that are perhaps less applicable to proteins. Interestingly, in this context, Roelfes and co-workers have reported that the Ir(lll)-mediated reaction of trifluoroboronates (4–10 equiv trifluoroborate salts, 0.1 equiv. [Ir{dF(CF_3_)ppy}_2_(dtbbpy)]PF_6_, 3 h, blue LEDs, r.t.) can allow one site (Dha16) to be selectively modified in the presence of not only Dhb but other Dhas in the natural product cyclic peptide thiostrepton (Figure 9).^64^ It was suggested that this was also, in part, guided by the presence of the neighboring thiazole group through its electron deficiency compared to other Dhas. By contrast, all of the Dha and Dhb sites in natural product cyclic peptide nisin were modified under essentially identical conditions, suggesting that a range of factors may contribute to this rare study of SOMO-phile selectivity in ncAAs. It remains to be seen as to how these may translate to selectivity in protein systems but they usefully highlight potential.

### Perspectives for off-Protein C• Radicals

“Off-protein” radical additions to proteins have provided a wide substrate scope with broad functional group tolerance. However, protein functionalization targeting canonical residues remains a challenge. Most carbon-centered radicals display a degree of nucleophilicity^73^ toward which many canonical AAs are unreactive. The primary exception, His, is inherently less susceptible to radical addition compared with reactive Trp and Tyr. As a consequence, selectivity is reduced.

The noncanonical residue dehydroalanine, by contrast, is a significantly more effective radical acceptor that reacts well with nucleophilic C-centered radicals, greatly diversifying the incorporated functionality that can be introduced and with essentially undetectable background reaction with any canonical AAs that may be present. Usefully, the site and number of such Dha tags can be controlled relatively well, allowing consequentially a much higher selectivity that therefore proves effective in C–C-bond forming protein editing. Nonetheless, limitations remain in, for example, the poor selectivity in current systems that leads to overall loss of configuration in a typical editing workflow l-[AA] → Dha → d/l-[AA’] (where [AA] = Cys, Ser, modified Sec etc; [AA’] = new residue of choice in a given protein). In a range of Dha reactions only modest d.r.s are obtained. Inherent substrate control over stereoinduction at Dha in peptide models is seemingly low unless neighboring residues with strong conformational bias generate local effects.^74^ Interestingly, suggestive examples can be seen in model systems that may allow engagement of backbone carbonyls in magnesium-mediated chelates bearing chiral bisoxazoline ligands.^75^ These, coupled with the longstanding utility of the Beckwith chiral methyleneoxazolidinones,^76^ suggest some feasibility that may be applicable to diastereoselective hydrogen atom transfer. These examples have largely been limited to organic solvents under nonrepresentative conditions, although there has been promising suggested utility under mixed aqueous systems.^77^ These systems all essentially exploit strong/cyclic constraints on local conformation to heighten diastereoselective substrate control, and it may be that alternative methods that exploit reagent/catalyst control might find utility in a background of weak substrate control.

Additionally, there are several important and useful functional groups that have not yet been successfully added to Dha in proteins at the time of writing, such as aryl radicals that could provide useful mimics for Tyr, Trp, Phe, and His (as well as their PTMs), although promising model systems^78,79^ have been usefully explored and accompanied by insightful mechanistic analyses^80,81^ as to the challenges and potential limits that currently exist, including the relayed use of off-protein radicals.^81^ The potential for combining utilization of off-protein radicals and the on-protein radicals that are generated as useful consequential intermediates (propagated) is highlighted by promising trapping of the alpha C• that arises from Dha in initial examples of dual Cα + Cβ functionalization. These include Cα–C in proteins^35^ and even, excitingly, Cα–F trapping in amino acid models^82^ (albeit the latter under nonaqueous conditions) and are discussed in additional detail in the following section.

## “On-Protein” Radical Approaches

Inverting the strategy described in the previous “off-protein” radical approach section provides a unique yet complementary alternative for exploiting the compatibility of C-centered radicals in protein modification (Figure 1b). One can envision utilizing either a reactive subset of canonical protein residues or leveraging previously installed reactive handles (via post-translational mutagenesis, genetic code expansion, or other methods) as radical precursors. Thus, an efficient and sufficiently mild radical initiation process would allow for the selective formation of C-centered radicals on the protein site of interest. This “on-protein” radical could then be utilized to react with a wide variety of “off-protein” radical acceptors to create C–C and C–heteroatom bonds, provided that those acceptors display sufficient chemoselectivity to not react with endogenous functional groups.

As noted above, early work on this concept was inspired by peroxidase-catalyzed oxidation reactions, furnishing thiyl and tyrosinyl radicals that produced largely uncharacterized protein dimers upon radical homodimerization.^36^ Later work focused on utilizing other native residues,^83^ and more recently, previously installed ncAAs possessing superior radical precursor abilities for radical generation, permitting a plethora of new applications in protein polymerization,^84^ bioconjugation,^85^ and protein–ligand interaction studies.^86^ While promising, the “on-protein” radical approach to protein modification faces many of the same barriers as the “off-protein” radical approach, namely chemo-/site-selectivity, limited choice of radical precursors, ease of preinstalled reactive handle installation, mildness of reagents and conditions, and unwanted off-target reactivity. These considerations and others will be evaluated for every technique discussed below, and similarly split according to radical generation at sites dictated by canonical side-chains or radical generation on those dictated by noncanonical side-chains, including those that generate C• centered radicals in protein backbone as well as side-chains. Furthermore, each strategy will be evaluated in terms of the new abilities or applications they offer to protein chemistry, and their potential for future refinement and utilization.

### “On-Protein” Radicals Generated on Canonical AAs

**Figure 10:**
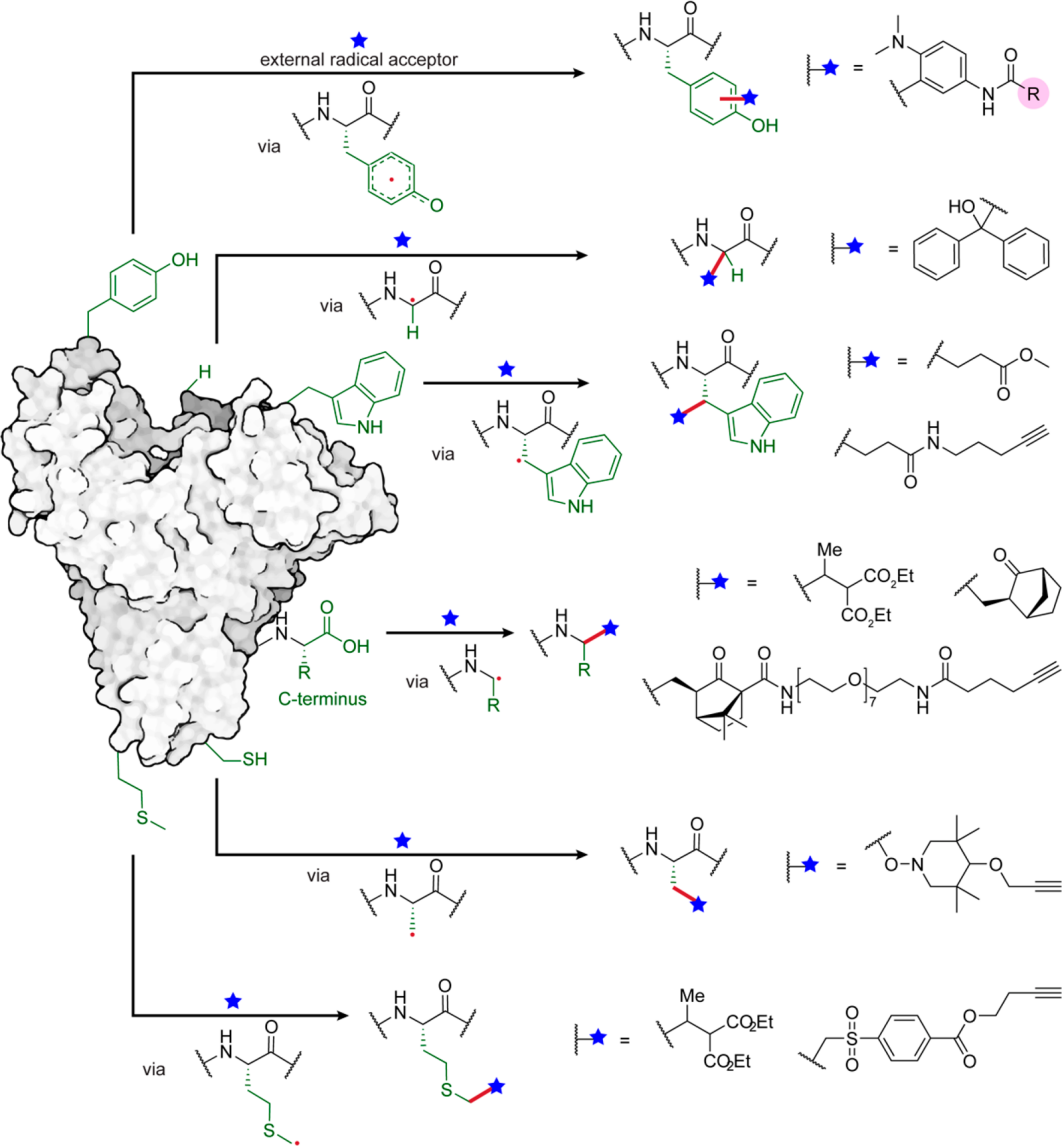
Overview of putative “on-protein” or “on-peptide” radicals generated on canonical AAs, including the C-terminus and different in-chain residues.

**Figure 11:**
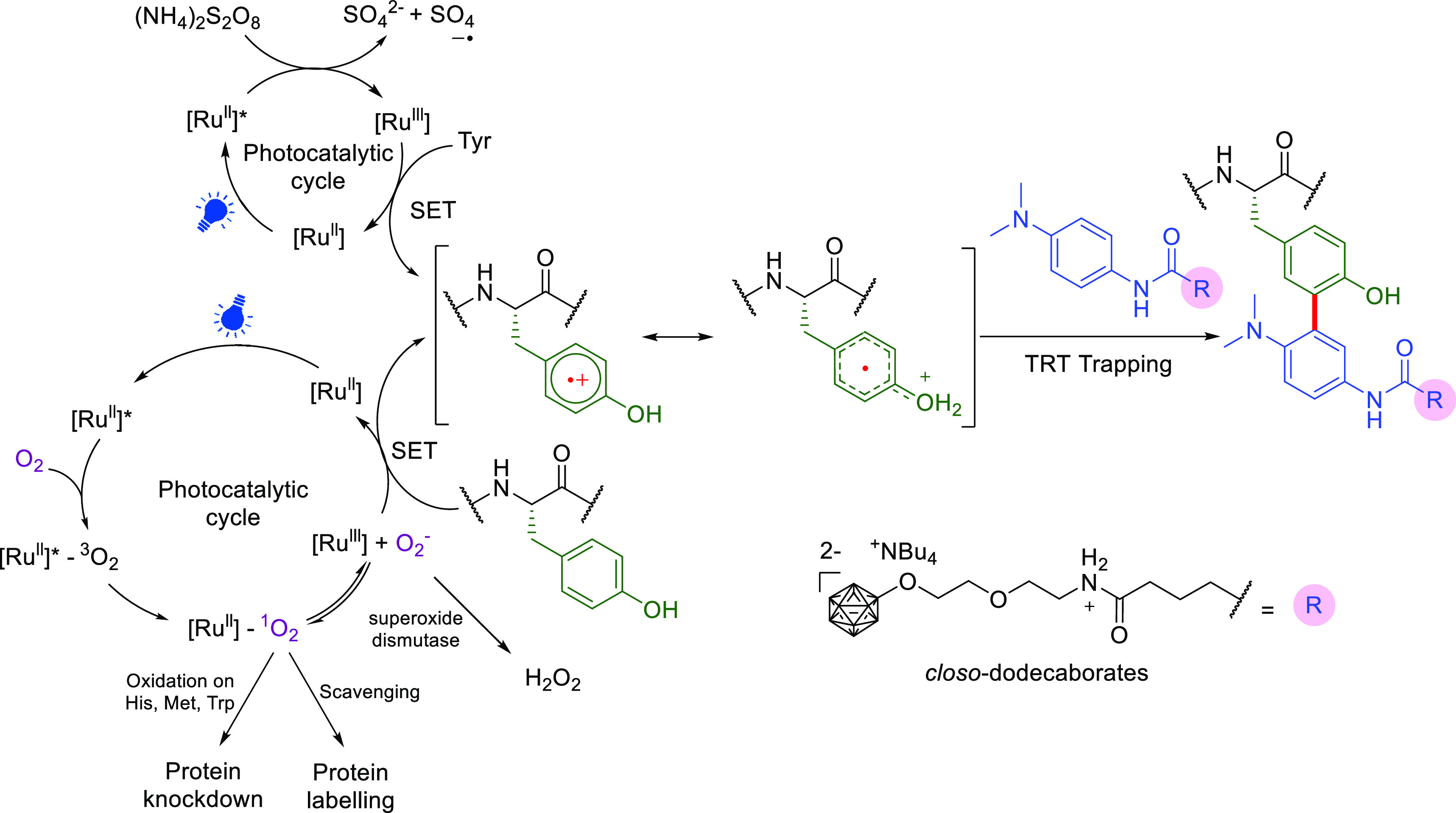
Oxidative radical generation on Tyr and a suggested mechanism that exploits dual roles of Ru(II) in inactivating or labeling proteins using on-protein generated tyrosyl radicals.^98−101^ Figure adapted from ref (100). Copyright 2015 American Chemical Society.

An ideal approach to “on-protein” radical generation would be through mild and selective radical initiation on a native protein residue, followed by a subsequent radical addition to an “off-protein” partner (radical acceptor) of interest (Figure 10). As a consequence of their electron-rich nature, several methods have been developed for the oxidative radical initiation of Tyr and Trp residues in proteins and consequently dominate examples in C• centered protein chemistry. Other, more rare, approaches (e.g., Gly, Met, and Cys) are complemented by the potential for exploiting the unique nature of termini also as canonical sites (e.g., decarboxylation of the peptide C-terminus) (Figure 11).

#### Tyr-Derived Radicals

In biological environments, the single-electron-oxidation of tyrosine residues can be usefully achieved, using a co-oxidant such as hydrogen peroxide, and technologically exploited through the use of several metalloenzymes, including peroxidases,^87−89^ tyrosinases,^90−92^ and laccases.^93−95^ Some but not all of the resulting conjugations exploit C• chemistries; aspects of these have recently been reviewed elsewhere^96,97^ and useful biomimetic parallels already drawn,^39^ and so we will not cover these in further detail here.

We have noted in the introduction, the prescient use of photoredox catalysis in generating on protein tyrosinyls.^37^ This method has since been further adapted via localization/proximity effects^51^ through prior conjugation of photocatalysts to putative protein ligands/partners. In this way, as well as exploiting the long-known chemoselectivity for electron-rich aromatics (phenolic or anilinic) as partners, reaction control that promotes enhanced generation of an on-protein tyrosinyl radical of interest can exploit photocatalysts localized onto a protein region of interest. For example conjugation to a known protein ligand enhances generation of radicals on proximal Tyr residues to create a form of regioselectivity. Moreover, it can be used to target a given protein among others. For instance, a benzenesulfonamide-linked Ru(II) photocatalyst has been used in a method (0.4 equiv modified Ru(ll) photocatalyst, 200 equiv TRT, visible light (>420 nm light source), 15 min, 0 °C, pH 6.0) that is designed to localize C• radical generation to carbonic anhydrase (CA) to selectively modify it using various acceptors (so-called tyrosine radical trappers (TRTs) such as *N*-acyl phenylenediamine). This is a localization that appears to function even within intact mouse erythrocyte lysates (where CA is abundant).^98^ It can also be applied concomitantly with affinity purification (of CA and dihydrofolate reductase (DHFR) from HeLa cell lysate) through the use of Ru(ll) photocatalyst-loaded affinity beads bearing appropriate ligands to allow simultaneous protein labeling purification and labeling with biotin-TRT.^99^

In these methods, mechanistic studies suggested that singlet oxygen generation by Ru(II)* can be used as an alternative to other oxidizing reagents (e.g., ammonium persulfate) as co-oxidant in the key Ru(ll)*/Ru(III) photocatalytic cycle (Figure 11). Based on this finding, elegantly modulated inactivation (via ^1^O_2_) vs labeling (via Ru(III) C• Tyr generation + TRT reaction) of epidermal growth factor receptor in A431 cells was accomplished by controlling the addition of various TRTs (2 equiv modified Ru(ll) photocatalyst, 200 equiv TRT, visible light (>420 nm light source), 30 min, 0 °C, pH 6.0).^100^ In the absence of TRT, only His, Trp, and Met in specific regions of the CA close to the catalyst-binding site were oxidized by the generated ^1^O_2_ in a mixture of CA and bovine serum albumin (BSA), leading to CA inactivation. In the presence of TRT-mediated labeling concomitant scavenging of singlet oxygen species reduces unwanted background oxidation. Extension to other conjugates can be achieved using altered TRTs: *closo*-dodecaborate derived TRT (TRT-DB) was conjugated to Tyr in BSA^101^ (100 equiv Ru(bpy)_3_Cl_2_, 100 equiv TRT-DB, 100 equiv ammonium persulfate, UV light, 1 min, 0 °C), yielding a system for proteins that might be a model for boron neutron capture therapy.

#### Trp-Derived Radicals

A clear reduction in reactivity is observed for the other canonical residues. The least abundant essential AA in nature,^102^ Trp would be a logical target as this would allow good control of site-selectiity based on low copy number. However, the often-buried sites of Trp in protein cores can make them challenging targets as sites for reaction. This may explain, in part, the current limitation of C• radical methods essentially to only on-*peptide* radical generation. Several examples highlight the strong potential for chemoselectivity but also associated challenges.

**Figure 12:**
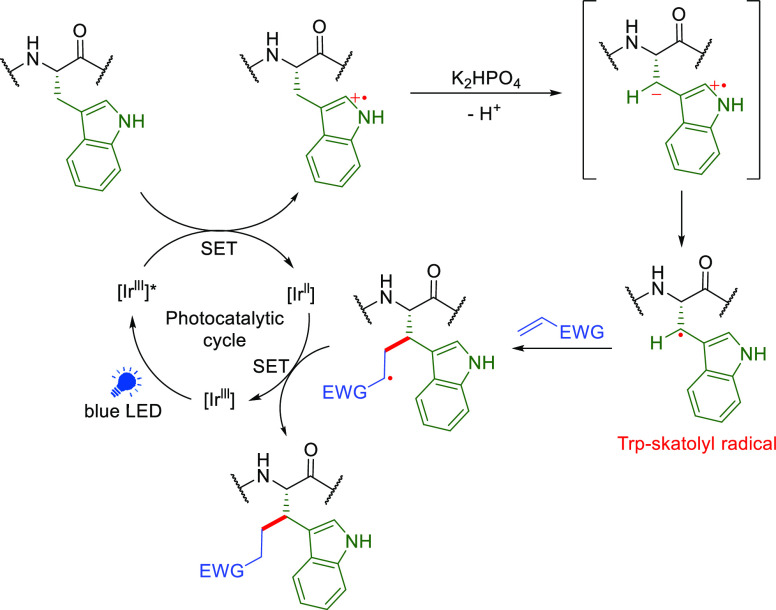
Mechanism of photocatalytic bioconjugation between a Michael acceptor and the β-position of Trp.^103^ Figure adapted from ref (103). Copyright 2018 American Chemical Society

Merck have reported an interesting photocatalytic on-peptide radical generation at the Cβ of Trp residues in glucagon and GLP-1 peptides using mixed buffer/DMF solvent systems, allowing reaction with external trapping acceptors (Figure 12).^103^ In the suggested pathway, upon photoexcitation, a nitrogen radical cation in the indole ring is formed via SET from the excited Ir(lll) catalyst, here Ir[dF(CF_3_)ppy]_2_(dtbbpy)^+^PF_6_^–^. Concomitant deprotonation of the β-carbon by base to afford a more stable Trp-skatolyl radical creates a neutral C• that may be trapped by radical acceptors. Under these conditions in certain peptide substrates the excess of trap that is also a conjugate electrophile (e.g., methacrylate) led also to N-substituted products via Michael-type conjugate additions at His and Lys residues.

Trp radical cations have also been invoked as putative intermediates formed from *in situ* photoexcited electron donor•acceptor pairs (Trp donor•Katritzsky-type pyridnium acceptors) that allow elegant methodology for Trp reactivity under a range of wavelengths.^104,105^

#### Met-Derived Radicals

**Figure 13:**
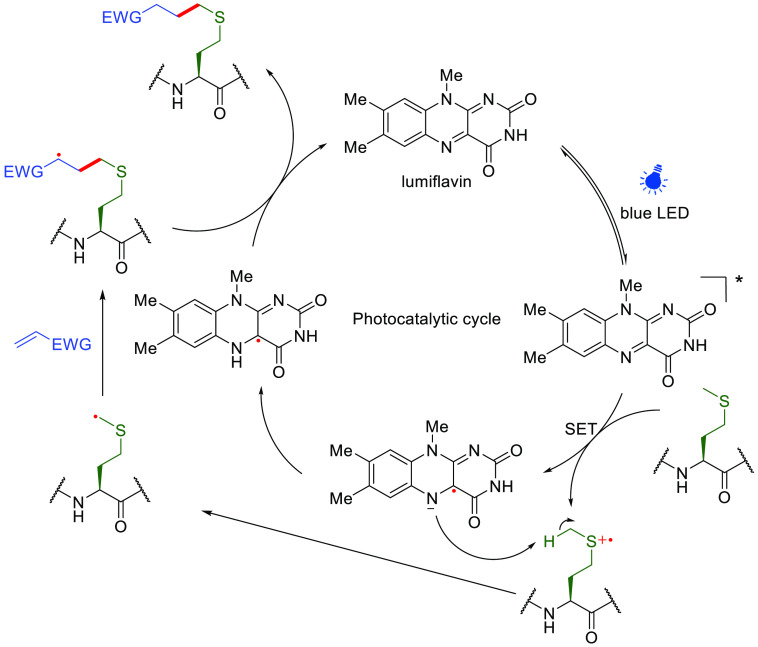
Proposed mechanism of photocatalytic methionine bioconjugation via Met-derived Cε• radicals.^112^

Met is also of relatively low abundance within the protein structures.^106^ While long-standing Met alkylation methods^107^ have been expanded of late,^108−110^ generated products or intermediates may lack stability, and indeed the displacement of Sδ cyanosulfonium is the basis of the classical cyanogen bromide backbone cleavage method.^111^ Recently, MacMillan and co-workers have demonstrated photocatalytic methionine bioconjugation in a range of protein substrates, including myoglobin, human growth hormone, ribonuclease A and CA (10 equiv lumiflavin, 200 equiv Michael acceptors, 450 nm, 30 min, pH 7.4) (Figure 13).^112^ Upon action of the excited photocatalyst, sulfur is proposed to undergo single-electron oxidation in Met, followed by concomitant α-deprotonation to give a Cε• radical at the Met methyl site in a manner akin to those at Trp described above. This generated Cε• carbon-centered radical could then react with various acceptors, furnishing the final conjugate product via HAT from reduced lumiflavin. Retention of relative fluorescence intensity in enhanced green fluorescent protein (EGFP) suggests reasonable biocompatibility, although competing Cys trapping was also noted (attributed to thiyl radical formation) with certain SOMO-phile/Michael-type acceptors. Consistent with observations of reactive accessibility (see above), the degree of Met surface exposure appeared to govern the distribution of sites of functionalization.

#### Cys-Derived C• Radicals

On-protein thiyl S• radicals generated on Cys side-chains are prevalent in essential biological processes^113^ and have been used to functionalize proteins^114^ via reaction with external radical traps. As a consequence, pathways that seek to exploit Cys derived C• face challenges of highly feasible competing radical pathways via unwanted S•.

**Figure 14:**
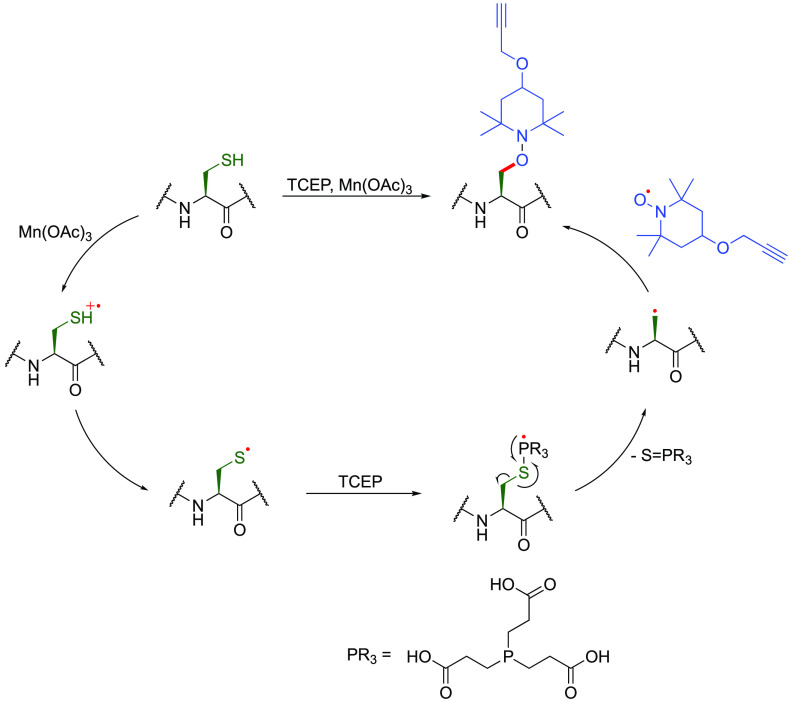
A possible mechanism of TCEP-mediated desulfurization of Cys, via thiyl radicals,^115^ forming C• alanyl radicals that can be trapped by TEMPO derivatives.^120^

Interestingly it has been proposed for over 60 years that C• alanyl radicals may be intermediates in observed Cys desulfurization reactions.^115,116^ Such desulfurizations are exploited in so-called “traceless native chemical ligation”^117−119^ to convert Cys to desulfurized Ala residues. In peptidic systems alanyl-radicals generated in this way have shown exciting promise by taking similar advantage of phosphine to activate the C_β_–S_γ_ bond.^120^ The presence of an alanyl radical could be promisingly inferred by trapping with TEMPO-derivatives in C–O bond formation (100 equiv TCEP, 20 equiv Mn(OAc)_3_, 5 equiv functionalized TEMPO-based traps, 2 h, 50 °C, pH 6.5) (Figure 14).^120^

Such reactions including prior strategies for desulfurization at cysteine, cystine, or selenenylcysteines proceed via a seemingly complex or possibly multiple-manifold process^121^ involving the likely intermediate formation of thiophosphoranyl radical adducts as precursors to C• radicals formed upon C–S bond homolysis via β-scission.^115,116^ The requirement in these systems for use of phosphines or other P(III) reagents, which are strongly reducing, will likely preclude general use in typical protein systems since these are commonly used to cleave disulfides. Indeed in “traceless” ligation methods oxidative buffering prior to refolding can be necessary, or, instead, the use of Cys-free targets is adopted. It has also been shown that eliminative mechanisms can compete via other phosphine-mediated desulfurization pathways.^68,122^ Nonetheless, these examples highlight possible strategies to enable C–S bond scission, for example from intermediate noncanonical amino acid precursors (see below, Figure 21),^123^ to allow access to on-protein alanyl radicals as productive intermediates.

#### Radicals Derived from Gly and Other Aliphatic Side-Chains

**Figure 15:**
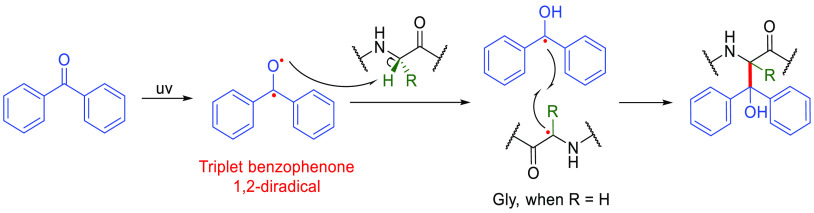
Mechanism of generating “on-protein” radicals via benzophenone derivatives.^128^

Notable early, near-classical, examples of on-protein C• radicals have involved the use of photolabile benzophenone derivatives in cross-linking,^124,125^ including the use of elegant ligand-directed methods.^126^ The high reactivity provided by the benzophenone diradical triplet excited state produced upon UV irradiation can abstract hydrogen atoms from the C–H bonds^127^ that are of course widely prevalent in proteins giving relatively stable ketyl radicals (Figure 15). The resulting on-protein carbon-centered radicals then rapidly recombine with the ketyl radical to form the desired bioconjugate. Peptide model systems have demonstrated some preference for the Cα–H of Gly residues,^128^ which may be attributed to the captodative effect of the resulting Cα• and by virtue of lower steric hindrance. However, the abundance C–H and the rapid recombination limits this strategy through the likely poor site-selectivity and restricted adduct scope, respectively.

#### Decarboxylation of C-Terminus

The C-terminus of a peptide or protein is an enticing target for site- and chemo-selective chemistry as it potentially sets itself apart from other highly abundant carboxylate containing residues (Asp and Glu). Its potential utility in proteins is shown by its popularity as a chemical handle in traditional esterification^4^ and amide coupling reactions,^129^ as well as through a redox-active ester (RAE) approach in decarboxylative peptide coupling strategies.^130^ Inspired by Barton esters,^131,132^ other RAEs such as *N*-hydroxyphthalimide^133,134^ and benzophenone oxime esters^135^ have also been identified to undergo a rapid radical fragmentation via SET during photocatalysis or transition-metal catalysis. With the aid of increasing reactivity revealed from RAE-terminated peptides, a wide scope of late-stage functionalization has been realized, including arylation,^136^ borylation,^137^ and alkynylation.^138^ Nevertheless, this methodology has to date been limited to short oligopeptides, in part due to the lack of selectivity with respect to unprotected side-chains (i.e., Asp and Glu), as well as the additional operational complexity, due to the required RAE conversion/generation step.^130^

**Figure 16:**
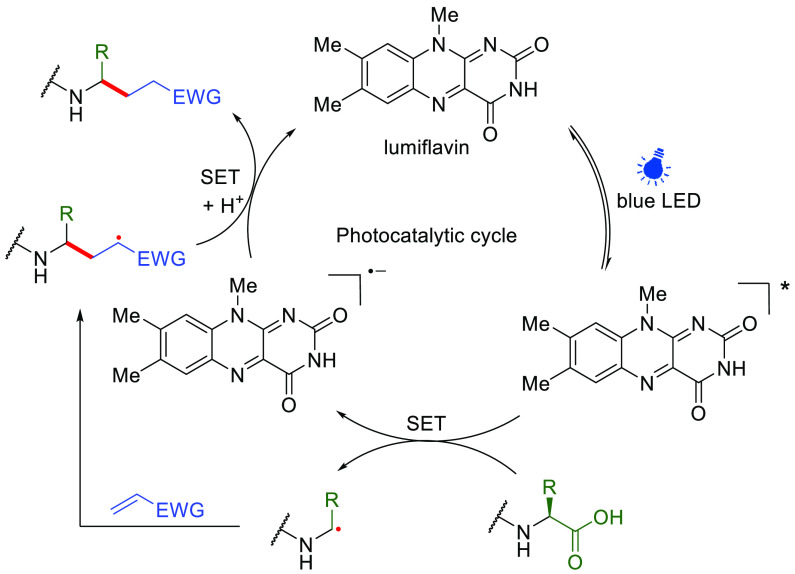
Proposed mechanism of site-selective bioconjugation via decarboxylative alkylation of the C-terminus.^85^ Figure adapted from ref (85). Copyright 2018 Nature.

MacMillan and co-workers accomplished a range of Ir(lll)-catalyzed peptide macrocyclizations utilizing a “naked” carboxylate C-terminus with an incorporated unsaturated AA as an intramolecular trap.^139^ One of the highlights was a demonstrated selectivity for C-terminal carboxylic acids over internal Glu side-chains in chosen peptide sequences. To better understand the selectivity, an elegant extended investigation suggested that this type of C• radical, resulting solely from C-terminal residues in native peptides, could be best induced by flavin-derived photocatalysts^85^ (Figure 16). This unusual selectivity was attributed to a narrow but effective difference in the oxidation potential (*E*_1/2_red ≈ 0.95 V (vs SCE (saturated calomel electrode)) for C-terminal carboxylates^140^ compared to *E*_1/2_red ≈ 1.25 V (vs SCE) for Asp/Glu^141^) arising from dative stabilization of the resulting Cα-carbon radical intermediates by the neighboring amide nitrogen. This allowed, when combined with suitable partner traps, generation of A-chain adducts on insulin (3 equiv photocatalyst, 10 equiv SOMOphile, blue LEDs, 8 h, r.t., pH 3.5). However, the somewhat extreme acidic conditions required to achieve this perhaps suggest that this selectivity may also exploit p*K*_a_ differences, and consequently a possible substrate-to-substrate subtlety. Additionally, as also noted above, “off-protein” radical acceptors are often also Michael-type conjugate acceptors. Selectivity for C-terminus C• radicals therefore also required fine-tuning through judicious use of appropriate traps. Interestingly, Trp-focused oxidative methods (see above) observed additional decarboxylative conjugation products instead at the B-chain terminus of insulin,^103^ further suggesting fine-tuned selectivity in this system.

### “On-Protein” Radicals Generated from Noncanonical AAs

The limitations for selectively and efficiently generating radicals on native residues in a full protein setting are being ably met through ingenious methods but face continued challenges on selectivity and compatibility. To overcome this, the engineering of superior radical precursors site-specifically into proteins provides an alternative, yet, come with the clear additional strategic burden of the need for methods for their insertion, whether those be biological or chemical. In this context, there is a somewhat blurred boundary between this section and the one above in that some of the ncAAs considered here are derived from canonical amino acids.

**Figure 17:**
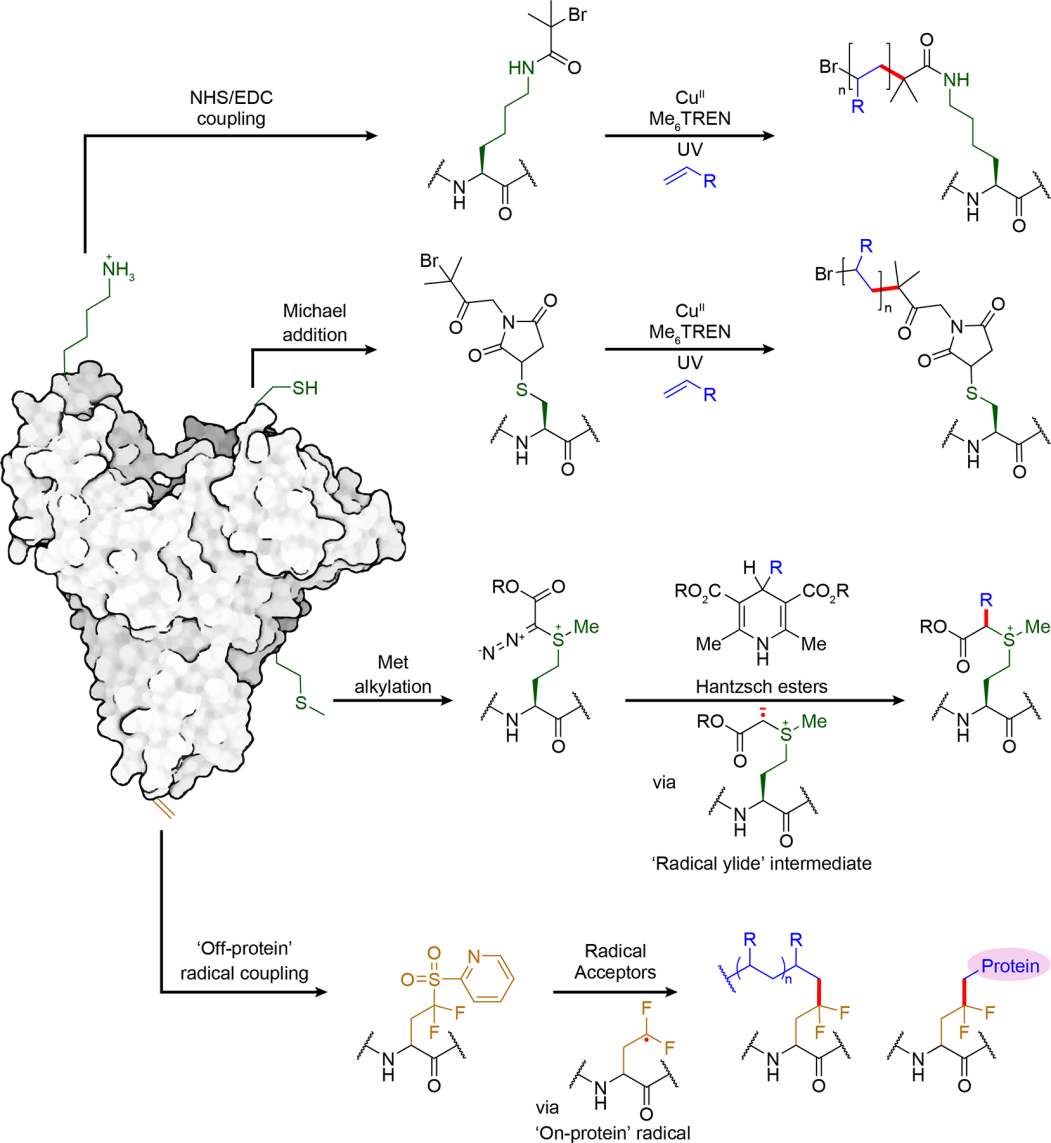
Overview of “on-protein” radical generation on noncanonical AAs.

Installation of predetermined functional groups of course ensures better control of targeted radical initiation, thereby potentially improving upon the lack of selectivity and reactivity of native protein residues and necessarily opening a window of selectivity beyond them to allow compatibility in endogenous frameworks (Figure 17). Many previous examples have adopted a “prosthetic” approach via preincorporation of radical precursors into proteins that are essentially disparate sites that allow a platform for known radical methods, for example radical polymerization.^142^ Their introduction has largely taken advantage of more traditional two-electron/heterolytic coupling strategies, for example using nucleophilic side-chains, in what might be termed residue-selective prosthetic bioconjugates. Conceptually, their subsequent C• radical chemistries are therefore perhaps ones that could be considered as, for example, polymerization platforms that happen to have a protein attached to them. There is clear and obvious merit in this given the role of some polymer bioconjugates in modified therapeutic proteins.^143^ These have been well covered elsewhere,^144−148^ and we will instead focus here on examples that aim to move past this prosthetic conceptual separation, although some are illustrative of broader roles of C• radicals on proteins, and selective examples have been chosen. Of the other techniques featured, many have explored alternative installation strategies for radical precursors that may therefore have potential well beyond the separate properties of “protein and platform”.

#### Prosthetic Attachment for Initiation at “Traditional” Sites (Lys, Cys)

As noted, an approach that has been commonly utilized in the construction of protein–polymer conjugates is one of attaching a prosthetic and “polymerizing-form”, where the prosthetic acts as the initiating site. “On-protein” radicals generated at premodified sites bearing prosthetic platforms (e.g., Lys or Cys coupled with radical precursor motifs) are then used for propagation with “off-protein” radical acceptors to afford the polymer chains.^84^ Commonly used radical-mediated polymerization techniques are centered on atom transfer radical polymerization (ATRP) and reversible addition–fragmentation chain-transfer polymerization (RAFT). Hybrid biomolecule conjugates are intended to address some limitations exhibited by native proteins (solubility, stability, circulation lifetime, as well as immunogenicity^145^) by introducing a coat of polymers with alternative, advantageous physiochemical properties to the protein surface. These protein–polymer conjugates are of great interest in the field of material science, and are discussed in detail in several reviews.^142−145^ The obvious limitations in design are those of complex heterogeneity created by the combination of heterogeneity in site (e.g., via multicopy Lys modification) with polydispersity in the emerging polymers. As a result, an increasing focus of recent studies is toward site-specific methodology.^146^

**Figure 18:**
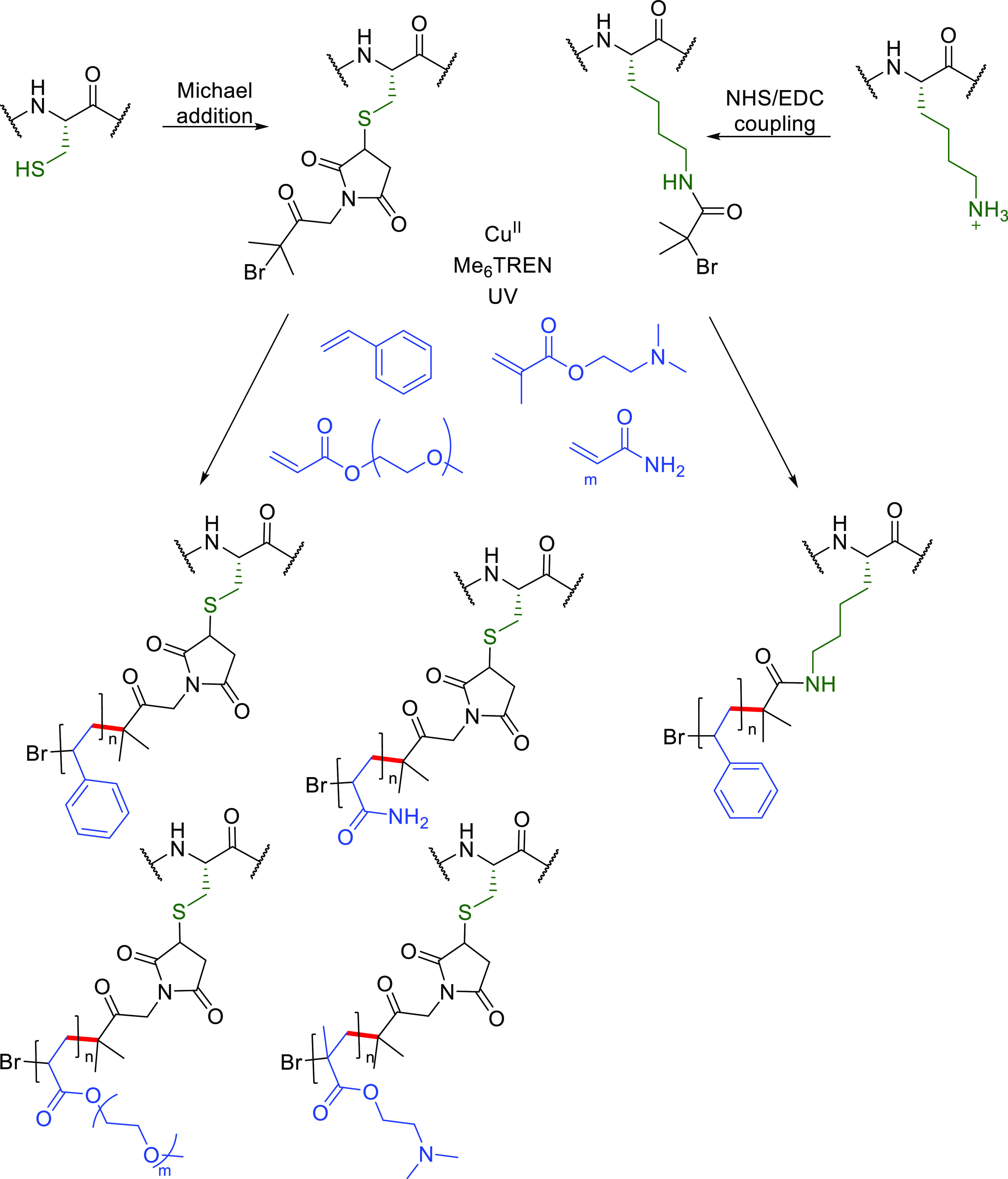
Construction of protein–polymer bioconjugates through a mild and oxygen tolerant photoinduced ATRP process.^149^

In these methods substrate scope has been restricted by a necessary presence of organic cosolvents or requirement for lower oxygen levels. Of interest is a mild, oxygen tolerant photoinduced ATRP process that allows use of both hydrophobic and hydrophilic monomers on several protein substrates in aqueous media (Figure 18) that shows some promise,^149^ applied thus far to models BSA, glucose oxidase, and β-galactosidase (500–2000 monomer, 2–12 equiv tris[2-(dimethylamino)ethyl]amine, 0.4–1.5 equiv CuBr_2_, UV or visible light, 3 h). Prosthetic, tertiary alkyl halide polymerization initiator platforms were preinstalled onto proteins via less selective, traditional Cys- or Lys-based methods (maleimide or EDC/NHS coupling) that bring with them complications of heterogeneity and/or linker stability. Nonetheless, relatively low catalyst loadings (Cu(ll) as low as 0.09 mM) proved possible, and removal of a light source provided a useful method for temporal control of the polymerization process that highlights the challenges and potential for future methods.

Recently, our group has demonstrated site-selective installation of a photolabile tetrafluoropyridyl moiety via Cys arylation (200 equiv perfluoropyridine, 30 min r.t., pH 7.4). This scaffold can then be photocatalytically activated to undergo desulfurization creating a stereoretained “on-protein” alanyl radical.^123^ These generated radicals were successfully trapped with various radical acceptors to create C–H, C–O, C–Se, C–B, and C–C bonds to give side chains with native l-amino acid stereochemistry (200 equiv radical acceptors or 1000 equiv B_2_Cat_2_, 100 equiv 4-methylbenzenethiol, UV, 60 min, 4 °C, pH 8.0).

#### Prosthetic Attachment for Initiation at Other Sites (Met)

**Figure 19:**
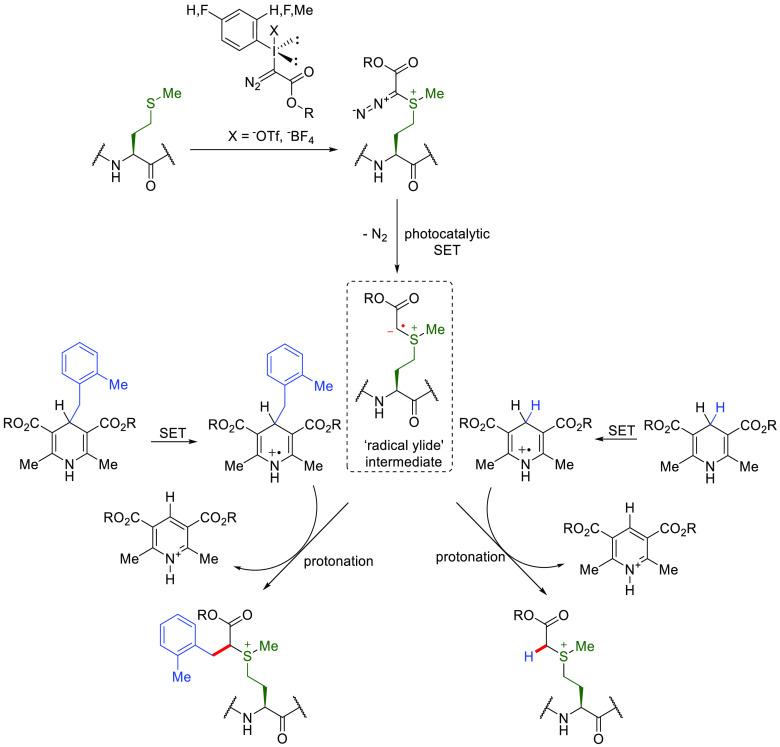
Proposed mechanism of consecutive Met-selective modification strategies that generates an “on-Met” C• radical ylid initiation site.^110^ Figure adapted from ref (110). Copyright 2018 Nature.

As discussed above, lower copy number residues provide better access to site-selectivity. Gaunt and co-workers utilized the strong electrophilic feature of an ethyl diazoacetate derived hypervalent iodine compounds to drive alkylation at Met to afford a sulfonium cation^110^ (500–1,667 equiv iodonium salt, 200–667 equiv thiourea, 50–167 equiv. TEMPO, 50–167 equiv formic acid, <5 min, 0–20 °C, pH ≈ 3) (Figure 19). Of greatest interest in this method is perhaps the utility of the resulting diazosulfonium product that can undergo further photocatalytic SET via a suggested radical ylid intermediate following liberation of N_2_. This type of on-protein C• allowed suggested (and exciting) radical–radical coupling products using several Hantzsch ester derivatives as precursors to the complementary “off-protein” radical.

#### Edited “On-Protein” Arylsulfones Allowing C–S Scission

**Figure 20:**
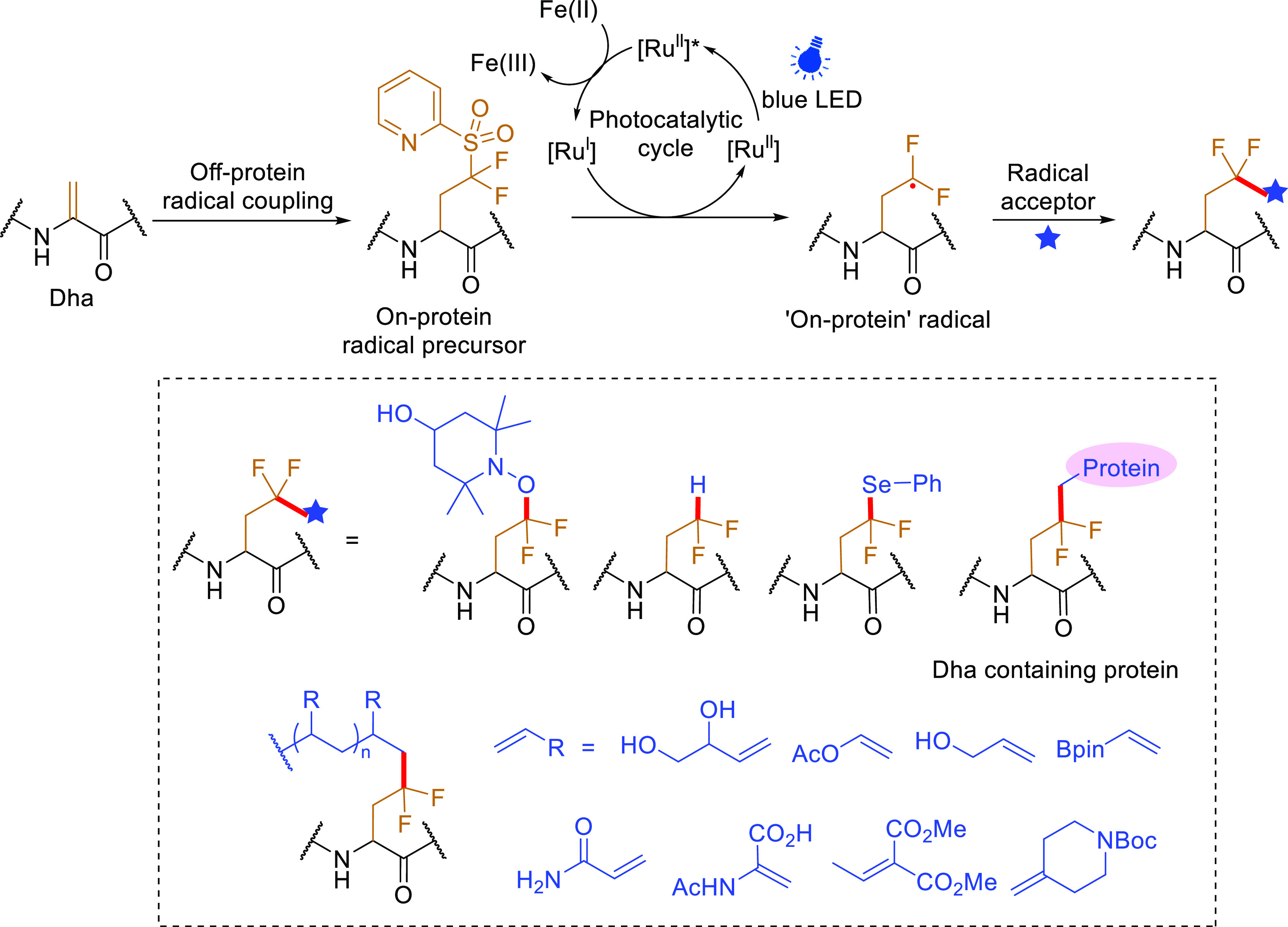
Proposed mechanism and use of photocatalytic generation of “on-protein” difluoroalkyl radicals from an installed “pySOOF” difluoropyridylsulfone and the resulting radical acceptor scope.^35^

Sulfones have displayed much potential as radical precursors in traditional organic transformations,^71,150^ and their use in on-protein methods could provide great flexibility given the precedent of biocompatibility seen for so-called pySOOF “off-protein” fluoroalkylradicals.^35^ Use of a bisfluoroiodosulfone enabled by C–I bond selective homolytic reductive initiation to create an “off-protein” •CF_2_SOOAr radical (see above, Figure 8) has allowed the creation of homogeneous site-specifically edited proteins bearing difluoropyridylsulfones as “on-protein” radical precursors. Further activation via reductive initiation gives an “on-protein” difluoroalkyl CF_2_γ• radical capable of reaction with various “off-protein” radical acceptors, typically with >90% conversion to desired products via C–C, C–Se, and C–O bond formation. The resulting adducts bear a minimal CF_2_ linker moiety between protein and acceptor allowing essentially “scarless” editing. Notably, despite the atypical nature of this radical site, oligomerization could be directly observed with suitable polymerizable monomers to synthesize “scarless” short-chain oligomer-protein conjugates creating future potential for protein–polymer conjugates where the polymer is essentially a direct part of the residue rather than resting on a prosthetic. Moreover, use of a protein radical acceptor allowed a dual “on-protein/off-protein” C• strategy in which an “on-protein” C• radical acts as the “off-protein” C• that is trapped by a protein SOMO-phile, here Dha. This enabled protein–protein conjugation based on trapping with another Dha containing protein (Figure 20).

#### Arylated Cys allowing C–S scission

**Figure 21:**
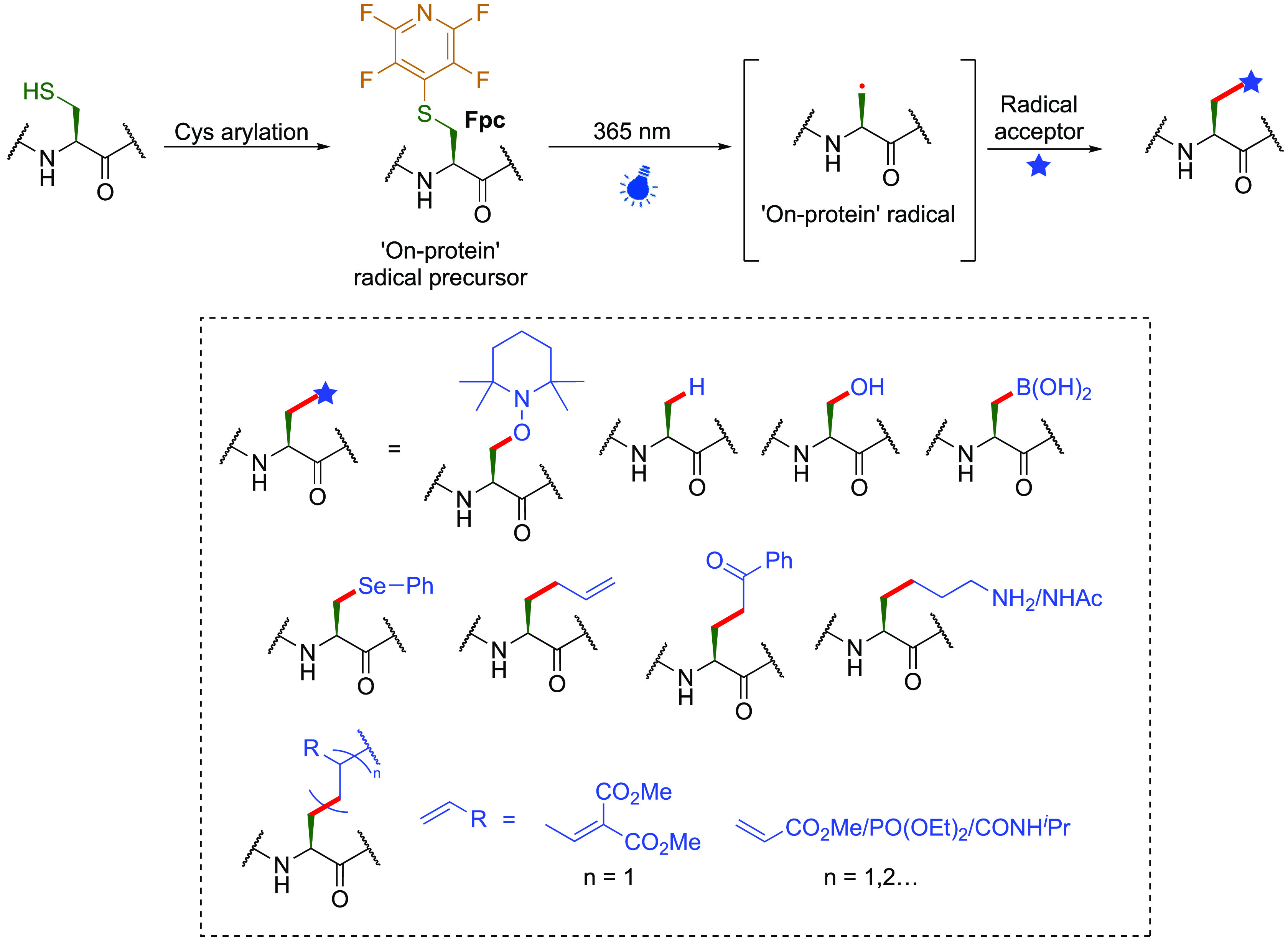
Exploitation of C–S bond scission in an arylated Cys noncanonical amino acid Fpc and alanyl radical trapping in a proposed method for so-called stereoretentive protein editing.^123^

This demonstrated potential of Cβ–Sγ scission in proteins via the creation of intermediates that allow effective trapping of on-protein side-chain radicals, coupled with known pathways for direct Cα–Sβ scission in Cys (see above), has prompted a recent study (Figure 21).^123^ Site-selective installation of a photolabile tetrafluoropyridyl moiety via direct S_N_2Ar Cys arylation to create pefluoropyridylcysteine (Pfc) (200 equiv perfluoropyridine, 30 min r.t., pH 7.4) appears to be highly selective and can be monitored advantageously via characteristic ^19^F protein NMR signals. This scaffold can then be photocatalytically activated to undergo desulfurization creating a stereoretained “on-protein” alanyl radical via apparent intriguing charge-transfer complexes (proposed to be ‘electron donor•acceptor’ (EDA) complexes). The use of appropriate reagent partners (200 equiv radical acceptors 100–200 equiv. arylthiol, 365 nm, 60 min, 4 °C, pH 8.0 or with 1000 equiv B_2_Cat_2_ for Cα–Bβ) thus generated radicals upon irradiation at 365 nm in buffer that were the successfully trapped with various radical acceptors to create Cα–Hβ, Cα–Oβ, Cα–Seβ, Cα–Bβ, and Cα–Cβ bonds. Resulting residues were shown to retain their l-configuration via diverse means (*in situ* NMR chemical shift perturbation, enzymatic processing, Marfey’s analysis^151^) and allowed the installation of some usefully functional side-chains in what is described as ‘stereoretentive post-translational protein editing’.

#### On-Protein, Postradical Addition/Propagated Radicals – “Post-Dha” C• Radicals

As noted in the “off protein” section, an interesting category of “on-protein” C• radical arises from the action of precursor “off-protein” C• radicals to create what may be considered as propagated intermediates (one radical intermediate generated from a prior). In “off-protein” Dha addition chemistry, long-lived benzylic radicals allow competing termination of the Cα• intermediate to create rare examples of Cα quaternary centers in protein backbones, here at a single difunctionalized (Cβ,Cα-dibenzylated) site. Given the role of such quaternary centers in peptide function^152^ the future potential of such sites in synthetic proteins is intriguing.

**Figure 22:**
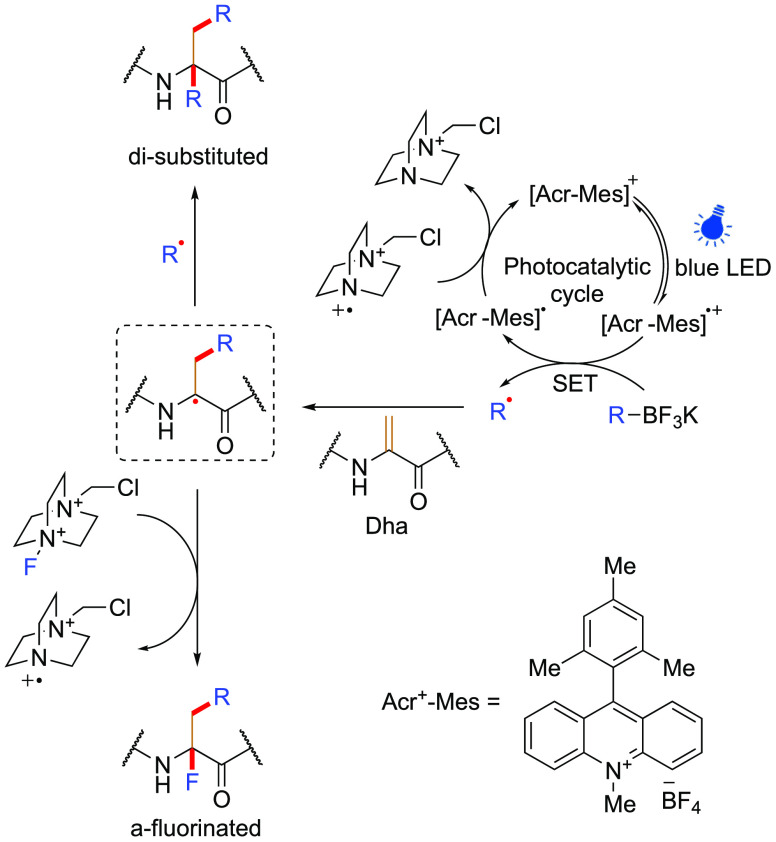
Mechanism of terminating a propagated “on-protein” α-carbon radical (dotted box) postradical addition to Dha. Multiple methods were demonstrated for initial “off-protein” C• formation, here, Acr-Mes was used as an example.^82^ Figure adapted from ref (82). Copyright 2019 American Chemical Society

Promise has also been seen in model systems of other Cα• trapping modalities from Dha precursors. Although restricted to amino acids, Molander and co-worker’s demonstration of the quenching of Cα• through C–F bond formation to generate α-fluoro-amino acids^82^ (2 equiv trifluoroborate, 5 mol % mesityl acridinium organophotocatalyst, and 4 equiv Selectfluor, blue LEDs, 12 h) shows exciting potential (Figure 22).

On-protein Cα• trapping has also featured in other intriguing applications. A suggested trapping of triplet oxygen at Cα• alanyl sites derived from treatment of Dha proteins with diboron(IV) reagents such as (HO)_2_B–B(OH)_2_ allows an efficient atypical protein cleavage mechanism that releases C-terminally amidated protein fragments via cleavage of the Nα–Cα.^153^ This “on-protein” trapping proves robust enough to be feasible even in cell lysates. In this way the controlled manipulation of propagated radicals becomes reminiscent of aspects of the uncontrolled effects of ROS upon proteins.^113^

### Perspectives for On-Protein C• Radicals

Carbon-centered on-protein radicals remain rarely used in comparison to other methods. This can be attributed to several current obstacles, including the lack of effective measures to achieve both high site-selectivity and desired efficiency by using stable and easily accessible reagents, driven in part by the limitations in inherent chemistries, primarily redox potentials, that are possible on native protein residues.

Of the methods available, several explore oxidative initiation of canonical residues raising immediate opportunities and yet challenges in “threading the needle” of varying half-potentials. The varied outcomes for the same radical precursor motifs, e.g. carboxylate, in different substrate systems under differing conditions highlight the challenges. Yet, if mastered, these offer immediate opportunities given the wealth of emergent methods.^154^

Direct routes to certain canonical sites would, by extension, likely have useful reactivity. The Cα• site of Gly is a propagated site in several methods and direct generation of a Cα• site that is rapidly trapped could offer opportunities. Some examples in small molecule systems offer a tantalizing sense of the potential and limits.^155^

New directions can be obtained from new functional groups. The premise of exploiting “on-protein” radicals using modified/ncAAs was in some senses proposed decades ago in the use of prosthetic initiation sites in polymerization.^147,148^ However, with increasing methods for generating integral “on protein” sites then the opportunity to control endogenous function (rather than perhaps tune the physicochemical) is enhanced; photolabile “on-protein” radical precursors in this regard allow useful temporal control.

The reaction of on-protein radicals also faces challenges in the competing selectivity of their off-protein SOMO-philes, especially in the propensity of typical radical acceptors for C• to also act as conjugate electrophiles for heteroatoms (and hence Cys, Lys etc.). Moreover, when simple functionalization is required, propagating oligomerization may also create an obstacle. Together the use of light intensity control coupled with the presence of effective quenching reagents (i.e., H atom source or coupling to other radicals) can halt unwanted radical propagation and so provide some measure of control.

## Conclusions and Outlook

Carbon-centered radicals are beginning to highlight a utility, mildness, and selectivity in protein reactions that one could argue was already hinted at by nature’s biosynthetic methods. While applications in more complex biological milieu still remain rare, promising compatibility is emerging; we have sought to highlight certain cellular applications above.^43,99,100,153^ They have proven to be especially well equipped to manipulate C–C bonds, a valuable disconnection in biology.^156^ While there are many elegant examples that target native protein residues, both on- and off-protein C• strategies have benefitted from the preinstallation of noncanonical side-chains possessing superior radical accepting or radical precursor qualities.

Moreover, the beneficial links to diverse photochemical methods, long applicable in radical initiation, but usefully extended by ever increasing photostimulated electron transfer methods are clear in a biological context and suggest diverse paths for implementation in increasingly more complex biological scenarios with control of reaction location and timing. Precisely matched photocatalyst “strengths” and irradiation conditions now can be considered as a benign pathway for desired radical reactivity via selective activation/initiation. Future developments of carbon-centered protein modification techniques should leverage this biocompatibility, spatiotemporal control, and tissue penetrating qualities of photocatalytic radical generation to explore protein modification chemistries *in vivo*.

Electrochemical radical generation strategies, while currently less explored and likely limited by aspects of implementation (electrode nature, adsorption, fouling etc.), offer a promising alternative as the redox “strength” may in principle be more finely controlled to match the generation of a desired radical reactivity at the residue of interest. Such control could prove crucial for development of further carbon-centered radical protein modification strategies, as many current methods use redox potentials at the extremes of “strength” generating ROS species and causing background oxidative protein damage that is sometimes overlooked. As for all synthetic methods in biological systems, in developing new radical protein modification strategies care must be taken to fully and transparently describe susceptibility to background oxidative damage (often in the form of Met oxidation), off-target reactivity, and general protein compatibility (by confirming the retention of protein structure and/or function after the reaction).

This review has focused more on synthetic methods than function, yet it is clear that if protein science is to move beyond mere conjugation (with dye, polymer or drug) to probe true function and selective, programmed access to that function—as a form of post-translational editing—then care will be needed. In this context, for example, sites that may provide convenient access may provide functional challenges. The C-termini of proteins for example are often relied upon to maintain protein structure^157^ and function;^158^ as for all sites this may impose limitations yet also offer unique opportunities—the key to exploring such function is likely overall to be determined by synthetic precision (linkerless, minimal alterations, discrete (*sic*) disconnections) rather than adherence to one synthetic method or another.

In summary, the potential to harness C• radicals in controlled methods, including precise protein editing, is now starting to emerge, and while many of the fundamental chemistries remain the same as those set down in Davies’ prescient two-decades-old review of protein radical chemistries^113^ our ability to harness them has perhaps moved them from perceived sites of damage to sites of “new function”.

## Supporting Information Available

Transparent Peer Review report available (PDF)
