# Supplementary material for: Carbon-Centered Radicals in Protein Manipulation
Source: ACS Cent Sci. 2023 Mar 21;9(4):614–38. doi: 10.1021/acscentsci.3c00051 (PMC10141601; doi:10.1021/acscentsci.3c00051)
Supplement: Supplementary file 1 — oc3c00051_si_001.pdf [file oc3c00051_si_001.pdf]

oc-2023-00051w.R1

Name: Peer Review Information for "Carbon-centred radicals in protein manipulation"

First Round of Reviewer Comments

Reviewer: 1

Comments to the Author

Davis et al are reviewing the current methods available to site-specifically modify proteins based on radical C-C bond formation. The manuscript particularly focused on the radical generation approaches and categorized these as on-protein or off-protein, and with/without the use of light and photocatalysis. This is an overall well-written and relatively comprehensive review that comes out right on time to reveal the emerging trend on radical chemistry-based post-translational modifications. The topic will certainly attract the audience and the manuscript is suitable for publication with several minor issues to be addressed below:

1. Regarding Tyrosine – targeted radical labeling, there have been recently several publications on the use of metalloenzymes such as peroxidase, tyrosinase, and laccase, etc. I would suggest the authors consider incorporate these papers and approaches into this review.

2. A lot of approaches summarized here were introduced in a way that chemical radical reaction can happen to a single protein or peptide target. How about the bio-orthogonality of these methods? It would be stimulating if the authors can comment more on whether certain methods can be selective to the target protein in the presence of other protein mixtures. Has any method been applied to or at least has the potential for applications in the cellular environment?

3. The authors mentioned “off-target incorporation into other amber codon sites” along with other considerations that could prove translationally prohibitive. Are there literature citations that can support these claims?

4. While most Figures are well polished and give great summaries of the approaches, the figure captions that explain these had better be referenced with corresponding literature citations related to these approaches.

5. Figure 17, the reaction products for the cysteine and the lysine-based conjugations appeared to have been mistakenly swapped.

6. The S(II/IV/VI) structure in Figure 5 looks confusing...

7. It reads better if “common two electron chemical approaches” in the Abstract can be changed to “common two-electron chemical approaches” . Also, “sidechain(s)” could be changed to “side-chain(s)” throughout the manuscript.

8. Page 3, line 44 “and, and” appears redundant.

9. Page 7, line 29- line 36, the sentence “For example, several current examples in protein modification are….. whilst simple aryl radicals …, something that may be attributed, in part, to the known….” Is long and reads a bit awkward. Please reword the writing here. Also, page 22, line 12-line 21 has a super long sentence.

Reviewer: 2

Comments to the Author

The manuscript covers an Outlook on the recent developments in utilizing radical-based intermediates for the selective modification of proteins. It is an evolving segment of an important research area and draws interest from diverse segments of Science. Hence, this Outlook is timely. The manuscript is well-written, clearly describing variables critical for developing a new method in this segment. The exemplification within “off-protein” and “on-protein” categories and subclassifications adds clarity. Hence, I think it would be suitable for publication in the ACS Central Science after the authors have addressed a few minor comments.

[1] (a) The title suggests that the Outlook would cover C-centered radicals in protein modification. However, there are examples where heteroatom-centered radicals in the bioconjugation reagent are involved.

(b) The abstract indicates that the Outlook will only cover radical-mediated C-C bond formation. However, it is not true as we move forward. There are cases of heteroatom engagement in the bond formation (e.g., Figures 13 and 18; related text).

Keeping these examples is a good idea as they add value. The authors can consider updating the title and the abstract to indicate the same. Alternatively, a disclaimer in the text could take care of it.

[2] The field is still in the early stage, and the authors have done a great job assembling the available examples. They might also consider looking at the paper on His-specific protein bioconjugation using visible-light-promoted C−H alkylation (J. Am. Chem. Soc. 2019, 141, 18230−18237).

[3] All the figures should be cited in the text.

Author's Response to Peer Review Comments:

Prof. B. G. Davis
Telephone: +44(0)1235 395008 or **+44(0)1865 275652**
Electronic-mail: Ben.Davis@rfi.ac.uk or **Ben.Davis@chem.ox.ac.uk**

Tuesday, February 7, 2023

Prof. Editor
ACS Central Science

Re: oc-2023-00051w

Editor,

Thanks indeed again for the invaluable feedback from you and the referees in your email of January 20th.

We now attach a revised, highlighted manuscript and associated files that we believe addresses all of the valuable issues raised, point-by-point, in the referees' and the editorial / formatting comments listed below. As well as addressing these comments, we have also taken the opportunity to add some recent additional examples to the review also.

Referee 1

Davis et al are reviewing the current methods available to site-specifically modify proteins based on radical C-C bond formation. The manuscript particularly focused on the radical generation approaches and categorized these as on-protein or off-protein, and with/without the use of light and photocatalysis. This is an overall well-written and relatively comprehensive review that comes out right on time to reveal the emerging trend on radical chemistry-based post-translational modifications.

• Thank you.

The topic will certainly attract the audience and the manuscript is suitable for publication with several minor issues to be addressed below:

1. Regarding Tyrosine – targeted radical labeling, there have been recently several publications on the use of metalloenzymes such as peroxidase, tyrosinase, and laccase, etc. I would suggest the authors consider incorporate these papers and approaches into this review.

• Thank you for this excellent suggestion. As the reviewer will know, this is a fundamental and wide area and so we have now referred to other, excellent reviews on the topic.

2. *A lot of approaches summarized here were introduced in a way that chemical radical reaction can happen to a single protein or peptide target. How about the bio-orthogonality of these methods? It would be stimulating if the authors can comment more on whether certain methods can be selective to the target protein in the presence of other protein mixtures. Has any method been applied to or at least has the potential for applications in the cellular environment?*

• This is an excellent question – thus far relatively few examples of been tested in more complex cellular environments or related lysates but there are some.
• We have highlighted some relevant examples in the main text and expanded in part on some of these in the revised version supplied here. We've also added a summary sentence to the conclusions that highlights this burgeoning application of C• centred radicals.
• Thank you for the suggestion.

3. *The authors mentioned "off-target incorporation into other amber codon sites" along with other considerations that could prove translationally prohibitive. Are there literature citations that can support these claims?*

• Yes indeed and we have added references to pertinent surveys and comparisons.

4. *While most Figures are well polished and give great summaries of the approaches, the figure captions that explain these had better be referenced with corresponding literature citations related to these approaches.*

• Thank you – yes, indeed – this is something that should have been present and we have now corrected this.

5. *Figure 17, the reaction products for the cysteine and the lysine-based conjugations appeared to have been mistakenly swapped.*

• Thank you for spotting this error !
• Now corrected.

6. *The S(II/IV/VI) structure in Figure 5 looks confusing...*

• Thank you – now corrected.

7. *It reads better if "common two electron chemical approaches" in the Abstract can be changed to "common two-electron chemical approaches" . Also, "sidechain(s)" could be changed to "side-chain(s)" throughout the manuscript.*

• Now corrected. Thank you.

8. Page 3, line 44 "and, and" appears redundant.

• Now corrected. Thank you.

9. Page 7, line 29- line 36, the sentence "For example, several current examples in protein modification are….. whilst simple aryl radicals …, something that may be attributed, in part, to the known…." Is long and reads a bit awkward. Please reword the writing here. Also, page 22, line 12-line 21 has a super long sentence.

• Thank you – both have now been rephrased and broken into shorter more tractable sentences !

Reviewer 2

The manuscript covers an Outlook on the recent developments in utilizing radical-based intermediates for the selective modification of proteins. It is an evolving segment of an important research area and draws interest from diverse segments of Science. Hence, this Outlook is timely. The manuscript is well-written, clearly describing variables critical for developing a new method in this segment. The exemplification within "off-protein" and "on-protein" categories and subclassifications adds clarity. Hence, I think it would be suitable for publication in the ACS Central Science after the authors have addressed a few minor comments.

• Thank you.

[1] (a) The title suggests that the Outlook would cover C-centered radicals in protein modification. However, there are examples where heteroatom-centered radicals in the bioconjugation reagent are involved.
(b) The abstract indicates that the Outlook will only cover radical-mediated C-C bond formation. However, it is not true as we move forward. There are cases of heteroatom engagement in the bond formation (e.g., Figures 13 and 18; related text).
Keeping these examples is a good idea as they add value. The authors can consider updating the title and the abstract to indicate the same. Alternatively, a disclaimer in the text could take care of it.

• Thank you for this question.
• In fact, prior Figure 13 (now 14) was present to illustrate the formation of the C• radical derived from precursors (including S•). The reviewer's question highlights our lack of clarity on the reason for its inclusion and we have now further emphasized this point in the text.
• Similarly in prior Figure 18 (now 19) we had sought to highlight the potential of radical ylids as radicaloids; the resulting reactivity here we feel is representative of C• radical chemistry. Again, the reviewer's question highlights our lack of clarity on the reason for its inclusion and we have now further emphasized this point in the text.
• We have also corrected the abstract as suggested.

[2] The field is still in the early stage, and the authors have done a great job assembling the available examples. They might also consider looking at the paper on His-specific protein bioconjugation using visible-light-promoted C−H alkylation (J. Am. Chem. Soc. 2019, 141, 18230−18237).

• Thank you – a nice example – now added as a section, along with an added Figure.

[3] All the figures should be cited in the text.

• Now corrected – thank you.

<u>Editorial Revision Checklist</u>

AFFILIATION 4: Please add a country to affiliation 4.

• Done.

TOC GRAPHIC: Please move the TOC graphic to the last page of the manuscript, beneath the REFERENCES.

• Done.

SYNOPSIS: ACS Central Science requires a brief synopsis. The synopsis should be no more than 200 characters (including spaces) and should reasonably correlate with the Table of Contents (TOC) graphic. The synopsis is intended to explain the importance of the article to a broader readership across the sciences. Please place your synopsis in the manuscript file after the TOC graphic.

• Done.

FIGURES: Please ensure that every figure is cited within the manuscript text.

• Done.

FIG. 13: Figure 13 includes a reference notation. Please confirm that this pertains only to data, and not the figure itself. If any of your graphics have been previously published this should be stated in the caption and copyright permissions should be obtained from any nonACS publications. Upload copies of the permissions as Other Files for Editors Only with your revision. Please note: If you have permitted use as the author of the previously published graphics, you do not need to supply written copyright permission.

• This pertains only to the data in this specific case.

Credit lines should read as follows: "Reproduced [or Adapted, Reprinted, etc.] with permission from Ref. [number], Copyright [year] [journal name]."

• Now added.

We look forward to hearing from you.

Yours sincerely,

Ben Davis,
on behalf of all authors.
